# High-Accuracy Detection of Neuronal Ensemble Activity in Two-Photon Functional Microscopy Using Smart Line Scanning

**DOI:** 10.1016/j.celrep.2020.01.105

**Published:** 2020-02-25

**Authors:** Marco Brondi, Monica Moroni, Dania Vecchia, Manuel Molano-Mazón, Stefano Panzeri, Tommaso Fellin

**Affiliations:** 1 Optical Approaches to Brain Function Laboratory, Istituto Italiano di Tecnologia, Genova, Italy; 2 Neural Coding Laboratory, Istituto Italiano di Tecnologia, Genova and Rovereto, Italy; 3 Neural Computation Laboratory, Center for Neuroscience and Cognitive Systems @UniTn, Istituto Italiano di Tecnologia, Rovereto, Italy; 4 Center for Mind and Brain Sciences (CIMeC), University of Trento, Trento, Italy

**Keywords:** two-photon imaging, GCaMP6, barrel cortex, neuronal ensembles, spatiotemporal neural responses

## Abstract

Two-photon functional imaging using genetically encoded calcium indicators (GECIs) is one prominent tool to map neural activity. Under optimized experimental conditions, GECIs detect single action potentials in individual cells with high accuracy. However, using current approaches, these optimized conditions are never met when imaging large ensembles of neurons. Here, we developed a method that substantially increases the signal-to-noise ratio (SNR) of population imaging of GECIs by using galvanometric mirrors and fast smart line scan (SLS) trajectories. We validated our approach in anesthetized and awake mice on deep and dense GCaMP6 staining in the mouse barrel cortex during spontaneous and sensory-evoked activity. Compared to raster population imaging, SLS led to increased SNR, higher probability of detecting calcium events, and more precise identification of functional neuronal ensembles. SLS provides a cheap and easily implementable tool for high-accuracy population imaging of neural GCaMP6 signals by using galvanometric-based two-photon microscopes.

## Highlights

• Smart line scanning (SLS) restricts imaging to the most informative image pixels
• SLS substantially increases the signal-to-noise ratio of population GCaMP6 imaging
• SLS leads to higher probability of detecting calcium events in population imaging
• SLS achieves more precise identification of functional neuronal ensembles

## Graphical Abstract

**Figure:**
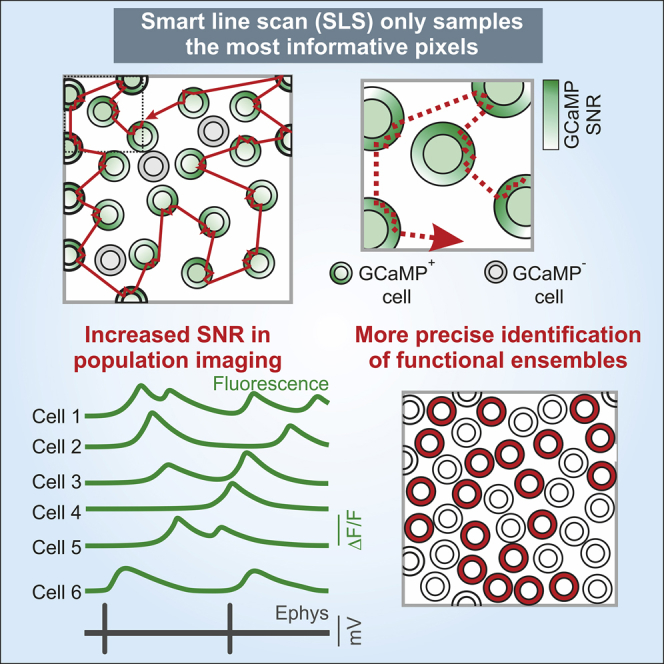

## Teaser

Using galvanometric mirrors and fast smart line scan trajectories, Brondi et al. present a method to significantly increase the signal-to-noise ratio in population GCaMP6s imaging. The method is validated in anesthetized and awake mice, and it leads to more precise identification of functional neuronal ensembles.

## Introduction

Spatiotemporal dynamics of neuronal activity underlie fundamental aspects of brain function, including the processing of sensory information, the generation of motor outputs, and the regulation of internal states (Harris and Thiele, 2011, Yuste, 2015). For example, sensory areas encode external stimuli varying their spatiotemporal dynamics (Grinvald and Petersen, 2015, Helmchen et al., 2018) in response to the presentation of different sensory stimuli. Various theories have been proposed to account for how these different activity patterns are used to extract sensory information that guides behavior (Okun et al., 2015, Quian Quiroga and Panzeri, 2009, Yang and Zador, 2012). Precise mapping of the fine spatiotemporal structure of these dynamics is, thus, of utmost importance to understand the cellular and network mechanisms underlying many aspects of brain function (Runyan et al., 2017).

Ideally, mapping neuronal activity at the population level should attain single-cell resolution, ability to detect single action potentials (APs), and robustness against artifacts (Harris et al., 2016). Currently, multiphoton fluorescence imaging is considered a preferred technique to this aim, enabling structure-function correlation and allowing single-cell precision over large populations of contiguous neurons (Dombeck et al., 2007, Helmchen and Denk, 2005, Ohki et al., 2005, Ouzounov et al., 2017, Stosiek et al., 2003). Moreover, the recent development of genetically encoded calcium indicators (GECIs) (Chen et al., 2013, Dana et al., 2016, Dana et al., 2019, Tian et al., 2009) allowed the use of bright fluorescent indicators with relatively high dynamic range and large quantum yield in genetically identified cell classes. Among these sensors, GCaMP6 is one of the most used because of its large dynamic range and high calcium binding affinity (Chen et al., 2013). When using raster scanning two-photon excitation to image at high acquisition frame rate small field of views (FOVs) containing one or few neural cells with high expression levels of the indicator, it is possible to record GCaMP6 signals with large signal-to-noise ratio (SNR). Under these optimized conditions, the detection of individual APs with high accuracy (>90%; Chen et al., 2013) can be achieved. However, the accuracy in detecting single APs and the SNR of GCaMP6 signals drops when the number of imaged cells increases (Harris et al., 2016, Huang et al., 2019, Theis et al., 2016) because the dwell time and, thus, the number of emitted photon per cell per unit of time decreases when imaging large FOVs. Moreover, heterogeneity in GCaMP expression may lead to variable levels of accuracy across cells, with low-expressing neurons having dimmer signals. Thus, *in vivo* population imaging of GCaMP6 with current raster scanning approaches systematically underestimates neural activities, missing neurons with low firing rates and/or low expression level of the indicator.

Here, we validated a method that maximizes the SNR of GCaMP6s over population of neurons. The technique uses image segmentation based on pixel-wise statistics and complex line scan trajectories (smart line scan [SLS]). Compared to raster scanning, SLS increased the SNR of calcium signals (average increase: ∼60%) and led to a higher probability of detecting calcium events over population of neurons (average probability increase: ∼36%) during spontaneous and whisker-evoked activities. SLS led to improved precision in detecting neural ensembles in layer IV excitatory neurons of the barrel cortex of awake animals. This method can be easily and inexpensively implemented in commercial raster scanning two-photon set-ups, allowing significant improvements in signal quality during population imaging *in vivo* with no change in the microscope hardware.

## Results

### SLS Samples Only the Pixels with the Most Information

**Figure 1:**
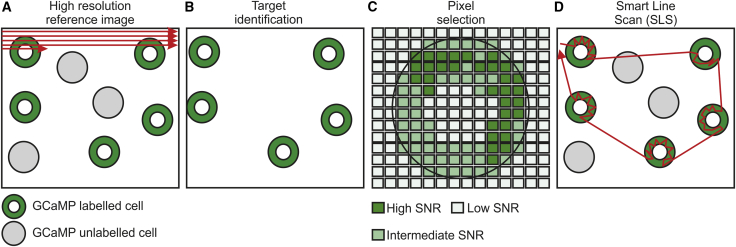
Schematic Representation of SLS (A) In raster scanning, images are generated scanning the excitation spot (red arrow) over all pixels in the FOV in a sequential raster trajectory. Fluorescence from GcaMP-labeled neurons (green), background structures (white), and GcaMP-unlabeled cells (gray) is sampled. (B) In SLS, scanned regions corresponding to labeled cells (green donuts) are identified based on the reference raster scan t-series. (C) Pixels belonging to one cell are sorted according to their SNR (green shades), and the subset of pixels maximizing the SNR is selected (ROIs). (D) SLS trajectories (red) intersect only selected pixels.

**Figure 2:**
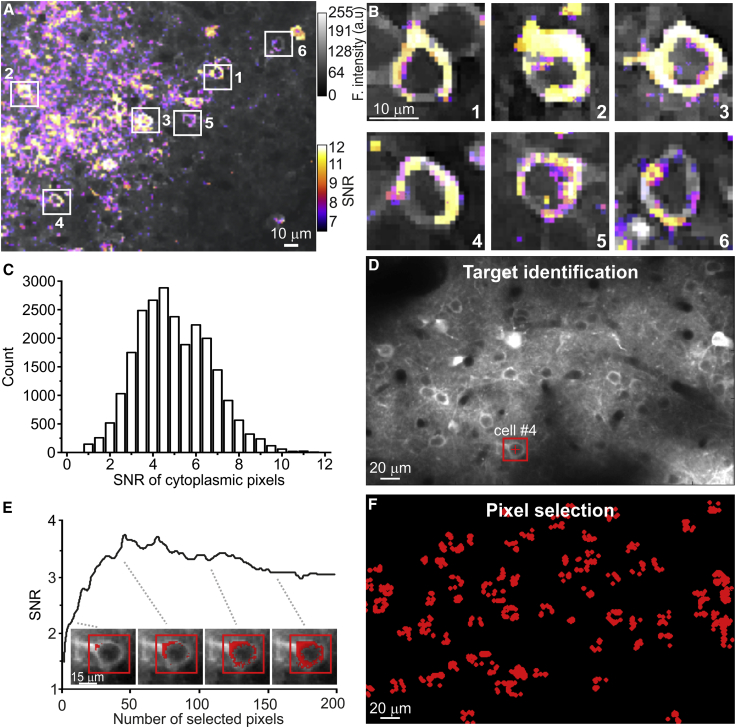
Pixel-Wise Statistics Identify the Subset of Most Informative Pixels to Scan Inside Each Cell (A) Two-photon raster scanning image showing layer IV neurons of the barrel cortex expressing GCaMP6s in an anesthetized mouse. Intensity projection of GCaMP6s fluorescence signal (gray scale) and SNR (pseudocolor scale) are shown. (B) Six cells in (A) are displayed at an enlarged scale. Color code as in (A). (C) Distribution of the SNR of cytoplasmic pixels (n = 164 cells from N = 8 mice). (D) Intensity projection of GCaMP6s signal (gray) of a t-series recorded in layer IV. The red cross indicates the center of the identified cell and the red box the boundaries of the region in which pixel-wise statistics are computed to identify ROIs. (E) Average SNR value as a function of the number of selected pixels for the cell showed in (D). Inset: four images of the cell highlighted in (D) are shown. Each image shows a different number of selected pixels (9, 46, 107, and 153, respectively). (F) Same FOV as in (D) after pixels selection. A total of 83 ROIs (red pixels) were identified.

**Figure 3:**
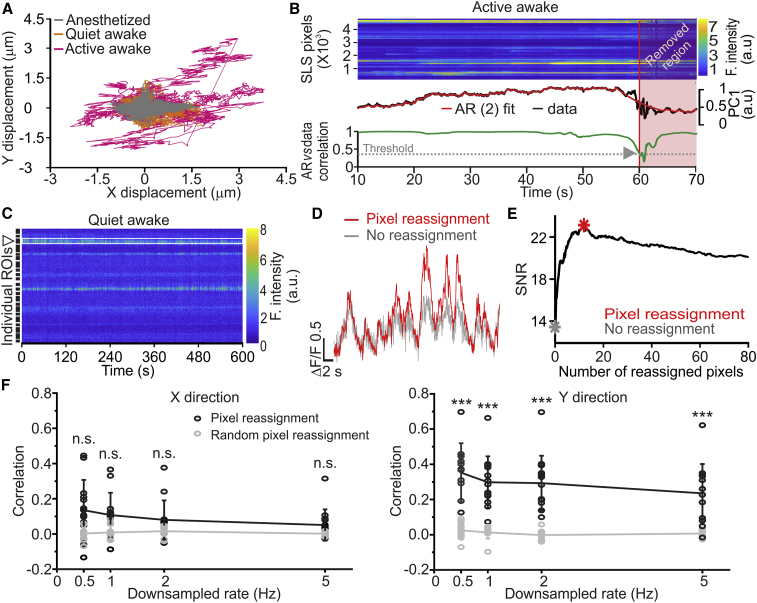
Motion Artifacts in SLS (A) X and Y displacement during raster scanning in anesthetized animals (n = 3 mice, gray), quiet wakefulness (n = 3 mice, orange), and active wakefulness (n = 3 mice, magenta). (B) Top: representative SLS acquisition during active wakefulness. Pseudocolor scale indicates fluorescence amplitude. Middle: autoregressive second order fit (AR(2), red) of SLS PC1 (black). Bottom*:* cross correlation (green) between AR(2) and the PC1. The gray arrowhead indicates a movement artifact detected when the 0.3 threshold is crossed. (C) Representative SLS acquisition with no detected large motion artifacts during quite wakefulness. (D) Fluorescence over time without (no reassignment, gray) or with (reassigned, red) the reassignment of 11 pixels. (E) SNR as a function of the number of reassigned pixels for one representative ROI. The gray and the red asterisks indicate the conditions displayed in (D). (F) Correlation between the motion displacement calculated using NoRMCorre on the reference patch and the motion displacement computed from the pixels reassignment approach (black dots). Empty circles: individual experiments; filled circles: average ± SD. Data from the reference patch were downsampled in time before applying NoRMCorre. Motion displacement values were separately calculated for the X (left) and the Y (right) direction in the reference patch and in the ROIs. The correlation between the motion displacement calculated using NoRMCorre and a random pixels reassignment are shown in gray. Left: downsampling 0.5 Hz, p = 0.08; downsampling 1 Hz, p = 0.18; downsampling 2 Hz, p = 0.06; downsampling 5 Hz, p = 0.32; paired t test, n = 13 SLS acquisitions. Right: downsampling 0.5, p = 2E-6; downsampling 1 Hz, p = 2E-4; downsampling 2 Hz, p = 2E-5; downsampling 5 Hz, p = 8E-9; paired t test n = 13 SLS acquisitions. In this as well in other figures: *, p < 0.05; **, p < 0.01; ***, p < 0.001; n.s., non significant. Error bars represent ± SD.

The workflow of SLS encompassed four main steps: (1) a high-resolution image of the target FOV was obtained using raster scanning (high-resolution reference image; Figure 1A); (2) target neurons were located (target identification, Figure 1B); (3) for each target cell, the more informative pixels were identified based on pixel-wise SNR statistics (pixel selection; Figure 1C); and (4) an optimized scanning trajectory, which sampled only the pixels of interest, was used to move the galvanometric mirrors (Figure 1D).

To test SLS, we used a dense and deep GCaMP6s staining in the mouse cortical layer IV (Figure S1). Using raster scanning, we acquired a first set of t-series in anesthetized animals (16 raster t-series from 8 animals; average frame period: 0.79 ± 0.17 s; average duration of raster t-series: 700 ± 288 s), and we asked whether SNR pixel-wise statistics could be used to improve the accuracy of GCaMP imaging. Figure 2A shows a representative SNR map (pseudocolor, see STAR Methods) superimposed to the GCaMP6s signal (gray) of a reference t-series. SNR values were heterogeneous across cells, with nuclear regions associated with low average SNR and cytoplasmic compartments with high average SNR (Figure 2B; average SNR in nuclear region: 1.4 ± 0.4; average SNR in cytoplasm: 3.6 ± 1.5; n = 8 FOVs from 8 mice). Within the cytoplasm, SNR values of individual pixels were variable, reflecting non-homogeneous GCaMP6s staining inside cells (Figure 2C; observed SNR distribution different from uniform distribution: two-sample Kolmogorov-Smirnov [KS], p = 3E-13).

For image segmentation, visual inspection was performed to identify the center of each cell of interest (point of interest, red cross in Figure 2D). A square box of lateral dimension similar to the cell diameter (red box in Figure 2D; average side dimension: 11.8 ± 0.7 μm, n = 604 cells from 8 mice) was centered on the manually identified points of interest. The dimension of the ensemble of relevant pixels for each cell, which we defined as region of interest (ROI), was determined by adding pixels within the box (red pixels in Figure 2E), starting from the ones with the highest SNR. In the majority of the cells (94%, 571 out of 604 cells from 8 mice), we observed that the average SNR within a ROI was an increasing function of the number of pixels (*n*) that reached a peak (average peak value: 5.0 ± 1.8, n = 571 cells from 8 mice) for *n* = 48 ± 32 pixels per cell (average total ROI surface: 182 ± 36 pixels; Figure 2E). Incorporating more pixels within the ROI tended to decrease the average SNR of the ROI (Figures 2E and 2F) because it included pixels from the nuclear and neuropil regions, which had lower SNR (average SNR in nuclear region: 1.4 ± 0.4; average SNR in neuropil: 2.0 ± 0.7; n = 571 cells from 8 mice). We compared the average SNR of ROIs identified with the semiautomatic method described above with the average SNR of manually identified ROIs, which included the cytoplasm and nucleus of those cells (average SNR in semiautomatic segmentation: 5.0 ± 1.8; average SNR of manual ROIs: 3.1 ± 1.5; n = 571 cells from 8 mice; two-sample KS test, p = 2E-50). Moreover, the number of pixels was smaller for ROIs identified based on pixel-wise SNR than manually drawn ROIs (pixel number in ROIs identified with pixel-wise SNR: 48 ± 32; pixel number in manually drawn ROIs: 75 ± 27; two-sample KS test, p = 2E-46). Based on these results, we decided to use the pixel SNR to identify ROIs and guide the generation of the SLS trajectory.

The SLS trajectory (yellow trace in Figures S2A and S3A) was built such that at each scan (1) the trajectory intersected all selected pixels in each ROI, (2) the trajectory intersected each ROI only once, (3) the trajectory first visited all pixels inside ROI_N_ then moved to ROI_N+1_ in a straight line, (4) the trajectory minimized (using a genetic algorithm) the total path length and the distance between the first and the last ROI in the sequence (see STAR Methods), (5) the pixels of the SLS were mapped as contiguous pixels in the 2D raster image, (6) the dwell time of the SLS was the same of that used in the corresponding raster image (4.4 μs in most experiments), and (7) a region surrounding each cell was scanned (surround region; Figures S3B–S3D). The dimension of the surround region was adjustable and pixels inside the surround region were mapped in a different data structure for separate analysis (see STAR Methods). The resulting SLS trajectory was then imported in the microscope software to control the movement of the galvanometric mirrors.

### Stability of Trajectory Computation and Precision of SLS

To evaluate the stability over time of pixel selection, we selected reference t-series (n = 8 from 4 mice) and divided them in shorter t-series, each one-fourth in length (62.5 s) compared to the original t-series (Figures S2A–S2B3). Trajectories obtained in short t-series largely overlapped (Figure S2C), and the average number of pixels was not different when SLS trajectory was computed on the long versus short t-series (6,513 ± 1,113 pixels for the 8 long t-series versus 6,547 ± 1,190 pixels for 32 short t-series, F-test, p = 0.23). The ratio between the number of selected pixels in short t-series and that selected in the whole series was on average 1.03 ± 0.25 (n = 8 long t-series and n = 32 short t-series, respectively), and the percentage of pixels showing 100% co-localization in all consecutive 4 short t-series for each long t-series was 74.0% ± 0.3% (n = 8; Figure S2E). Among pixels showing no co-localizations in consecutive short t-series, only a small fraction fell into ROIs (average: 0.21 ± 0.08, n = 8; Figure S2F). because the path length of a SLS depended on the heuristic solution to a nondeterministic polynomial time (NP)-complete problem (i.e., minimization of path length), small changes in pixel selection might affect the SLS duration. We found no difference in scan duration under the different conditions (average line scan duration for 8 long t-series: 0.049 ± 0.019 s; average line scan duration for 32 short t-series: 0.048 ± 0.004 s; paired t test, p = 0.77).

The actual movement of the galvanometric mirrors may lag behind the signal that controls them (command signal; Kummer et al., 2015). In many galvanometric mirror types, including the ones used in this study, the actual mirror movement can be monitored through the signal of the feedback output (feedback signal). To control whether the time lag between the command and feedback signal depended on the angle of the SLS trajectory, we generated line scans with regular polygon trajectories (Figure S4). Within each trajectory, the angle between successive segments of the polygon was kept constant. In contrast, trajectories differed for the shape of the polygon, such that different trajectories had different angles in the range 20°–160°, and there was at least 20° difference between the angles of two trajectories (Figures S4A and S4B). Importantly, this range included 96% ± 1% (n = 18 SLS trajectories) of the angles of a typical SLS trajectory. We measured the lag between the command and the feedback signals as the time of the peak of the correlation coefficient in the cross-correlogram. We found that the time lag was largely independent on the trajectory angle and was similar to the one observed during raster scanning (Figure S4D). We also found the time lag using SLS trajectories to be similar to that observed using the polygon trajectories (Figures S4C and S4D). The time difference between the command signal and the mechanical response of the galvanometric mirrors was incorporated as a time lag in the settings of the commercial acquisition software, and it was considered in the creation of fluorescence imaging data in both raster and SLS acquisitions.

To estimate the precision of mirror positioning (Figures S4E–S4H), we imaged a pollen grain by using raster scanning. We placed a SLS trajectory such that the convoluted portion of the SLS crossed the borders of the pollen grain. We plotted the intensity profiles of the SLS pixels in the portion of SLS trajectory crossing the pollen grain and compared this profile with the intensity profile of the same pixels acquired in the raster scanning image. The distance corresponding to the peak of the cross correlation between these two intensity profiles was taken as a measurement of the error in mirror positioning in SLS compared to raster scanning. We found this value to be <1 μm (Figure S4H).

We measured SLS trajectories acquired on a fluorescent grid at different acquisition rates (Figures S4I–S4K). The different SLS acquisition rates were obtained by changing the pixel dwell time from 4.4 μs to 1.6 μs in 0.4-μs steps. Fluorescence signals acquired in the range 3.6 μs–4.4 μs were highly correlated, whereas correlation decreased from those SLS acquired in the range 1.6 μs–3.2 μs (Figures S4J and S4K). This finding suggests that below a certain dwell time value (3.6 μs in our case), mirror positioning becomes less accurate.

Finally, we measured the reliability of mirror movements during SLS over time. We performed repetitive SLS on a grid (Figure S5) and found that transitions between fluorescent and non-fluorescent regions of the grid were sharp (Figure S5C), implying reliable and precise mirror movements across repetitions.

### Acquisition Rate versus Number of Imaged Cells in SLS

To evaluate the acquisition rate during SLS as a function of number of imaged cells, we first selected 292 cells in a densely GCaMP6-expressing cortical slice. We then identified subsets comprising 1–292 cells (1-cell increment), and for each subset, we generated four SLS trajectories with different surrounds (0, 1, 2, and 3 pixels, respectively). SLS duration was a linearly increasing function of the number of scanned cells and the slope of the linear fit was higher for SLS with increasing surround (Figure S6A). Moreover, the ratio between the pixels scanned in SLS and the pixel scanned in raster scanning decreased with the number of scanned cells (Figure S6B). As expected given a fixed number of cells to be scanned, the acquisition rate decreased with the dimension of the surround region (Figure S6C).

We then evaluated the performance of SLS on population imaging *in vivo*. GCaMP6s expression levels were heterogeneous across animals. We selected four experiments with a high level of expression (high < F > in Figure S6D) and four with low expression (low < F > in Figure S6D; see STAR Methods). The number of segmented cells in low-expressing mice was lower than in high-expressing ones (39 ± 19 cells, N = 4 experiments versus 135 ± 51 cells, N = 4 experiments; unpaired t test, p = 0.026). In contrast, the number of pixels required to maximize the SNR in each cell in low-expressing cells was higher than that in high-expressing animals (58 ± 11 pixels versus 36 ± 6 pixels, respectively; N = 4 experiments; unpaired t test, p = 4E-4). The acquisition rate was higher for low-expressing animals than in high-expressing animals (Figure S6D), indicating that the reduction in the number of segmented cells more greatly influenced the SLS acquisition rate than the increase in the number of pixels required to maximize SNR in low expressing compared to high-expressing animals.

### Motion Artifacts in SLS

Because SLS sampled only a subset of pixels without building an image of the sample, current methods for correction of motion artifacts could not be applied to SLS. We, thus, developed a strategy to identify and counterbalance motion artifacts in SLS. We first evaluated the average lateral displacement due to motion by aligning raster scanning t-series (maximizing frame-wise correlation) offline in recordings performed in anesthetized and awake animals free to run on a wheel. Sudden and large (≥3 μm) displacements of the FOV were observed upon running in awake animals, whereas during quite wakefulness displacements were small (<3 μm) and comparable to those observed under anesthesia (Figure 3A). We then developed a method to automatically detect offline the large motion artifacts associated with locomotion in SLS. An autoregressive linear model (AR(2)) was fit on the first principal component (PC1) of the whole SLS acquisition. When the correlation between AR(2) fit and PC1 dropped below a threshold value (set at 0.3), a large motion artifact was detected (gray arrowhead in Figure 3B). *A posteriori* visual inspection confirmed the presence of large, fast, and synchronous distortions of the SLS signal when AR(2)-PC1 correlation was <0.3. These distortions showed fast onset that were incompatible with GCaMP6 kinetics. All SLS acquisitions with AR(2)-PC1 correlation values below 0.3 (5 out of 26 line scans in 3 awake mice and 0 out of 22 line scans in 3 anesthetized animals) were discarded. The amount of detected large artifacts in awake animals decreased with the increase in the surround region, and they were never detected for surround values of 3 (0 out of 4 SLS acquisitions in 3 awake mice). Based on this evidence and the fact that movement artifacts were animal and preparation specific, we devised the following strategy to minimize the impact of these large artifacts on SLS. We first estimated the displacement associated with motion artifacts in a 10-min-long raster scan t-series. Based on the value of the observed displacement, a value of surround region was determined such that the scanned surround was larger than the observed displacement (with the scanned surround region smaller than the upper limit of 3 pixels). This strategy allowed us to decrease the occurrence of sharp discontinuities in SLS and to discard a minority of SLS acquisitions.

Besides large motion artifacts, smaller motion-induced distortions may still be present and confound the interpretation of the SLS signal. To partly compensate for these smaller artifacts, we processed the SLS acquisitions *a posteriori*, reassigning pixels to the cell regions or to the surround regions based on pixel SNR value in a sliding window of 10 s (the sliding window was moved in the temporal axis by one frame at a time). Pixels with higher SNR were assigned to the cell region even if they belonged to the initially assigned surround region (average number of reassigned pixels per ROI: 10 ± 8, n = 48 line scans from 3 awake and 3 anesthetized mice; Figures 3D and 3E). This strategy was based on the assumption that (1) the surround region was large enough such that it always contained the cell body of the recorded neuron despite the displacement due to the movement artifact and (2) the FOV displacement occurred in the X, Y direction. We observed that reassigning a limited number of pixels based on SNR values within each ROI along the SLS improved signal quality (Figure 3E).

We investigated the correspondence between the artifact readout strategy described above and the X, Y displacement simultaneously computed from the reference patch, a user-selected square box added at the end of the SLS trajectory (yellow box in the top right corner of Figure S3A). We tested whether pixel reassignment could be used to track motion. To this aim, we extracted the activity of the reference patch (in n = 13 SLS acquisitions) and we estimated sample motion in X and Y directions on the reference patch using non-rigid motion correction computed with NoRMCorre (Pnevmatikakis and Giovannucci, 2017). We computed the correlation between the shift obtained on the reference patch and the shift of the average position of the selected ROI pixels of the SLS trajectory (Figure 3F). Correlation values between the average Y position of ROI pixels selected with pixel reassignment and the Y shifts obtained by NoRMCorre on the reference patch were higher than correlation values between the average Y position of randomly selected pixels in the Y direction and the Y shifts obtained by NoRMCorre on the reference patch (Figure 3F, right). For the X direction, correlation of ROI pixels tended to be higher than those for randomly selected pixels, but the effect did not reach significance (Figure 3F, left).

### Neuropil Decontamination in SLS

**Figure 4:**
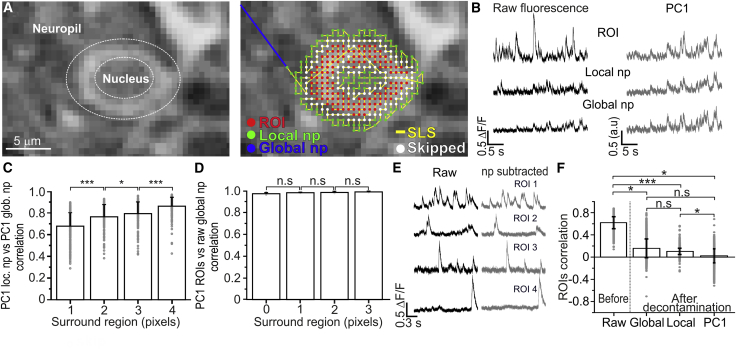
Neuropil Decontamination in SLS (A) Left: a GCaMP6s-expressing layer IV cell. Right: the pixels belonging to the ROI (red), the local neuropil (green, local np), and the global neuropil (blue, global np) are shown. SLS trajectory is shown in yellow and pixels at the border between the ROI and the local np were not considered (white, skipped). (B) Black traces on the left: raw fluorescence signal over time from SLS on one representative cell (ROI), its corresponding local np, and the global np. Grey traces on the right: PC1 computed from the combined pixels of all the ROIs (upper trace), PC1 of all local np regions (middle trace), and PC1 of the whole global np (bottom trace). (C) Pearson’s correlation between the PC1 of the signal from local np regions and the PC1 of the global np regions as a function surround region dimension (from 0 to 3 pixels). n = 172 ROIs from 11 SLS for each surround region value; paired t test, p = 1E-11 for surround 1 pixel versus surround 2 pixel; p = 0.04 for surround 2 pixel versus surround 3 pixels; p = 2E-9 for surround 3 versus surround 4 pixels. (D) Pearson’s correlation between the PC1 of the ROI signal and the global np as a function of the surround region dimension (from 0 to 3 pixels). n = 90 ROIs from 5 SLS for each surround region value; paired t test, p = 0.1 for surrond 0 pixel versus surround 1 pixel; p = 0.1 for surround 1 pixel versus surround 2 pixels; p = 0.96 for surround 2 versus surround 3 pixels. (E) Left: raw fluorescence over time for four ROIs (black traces) acquired in SLS. Right: corrected fluorescence signals for the same four ROIs shown on the left after neuropil decontamination (gray traces). Neuropil correction is obtained subtracting from the raw fluorescence traces the weighted PC1 of the global np signal. (F) Pearson’s correlation across ROIs before (raw) and after neuropil decontamination using different strategies (global np subtraction, local np subtraction, and subtraction of the weighted PC1 of the global np). n = 3,656 ROI pairs from 11 SLS; Wilcoxon rank-sum test, p = 0.02 for raw versus global np subtraction; p = 2E-4 for raw versus local np subtraction; p = 0.02 for raw versus PC1 of the global np; p = 0.08 for global versus local np subtraction; p = 0.07 for global np versus PC1 of the global np subtraction; p = 0.01 for local np versus PC1 of the global np subtraction.

For neuropil decontamination, we first defined two different types of neuropil signals (Figure 4A). The “local” neuropil of a given cell was defined as the average fluorescence signal present in the surround region of the SLS trajectory for that cell. The “global” neuropil was instead defined as that signal arising from all pixels of the SLS trajectory that did not belong to ROIs or surround regions, based on offline segmentation of the reference scanning image which was used to build the SLS trajectory (see STAR Methods). We calculated the correlation between the average PC1 extracted from each local neuropil region with the PC1 obtained for the global neuropil. We observed a strong correlation between the two principal components (Figures 4B and 4C) for all values of surround regions investigated, indicating that the local and global signals contain very similar neuropil information. Similarly, we observed a high correlation between PC1 of the ROIs and the global neuropil (Figure 4D), suggesting that the PC1 of all ROIs may be interpreted as a global neuropil under our experimental conditions. We then subtracted the global, the local, and the global neuropil signal appropriately corrected for the activation coefficient of each ROI (obtained from the principal-component analysis [PCA]) from the average fluorescence signal of each ROI. After this procedure was performed, we found that the correlation between the signals of different ROIs was reduced (Figures 4E and 4F). Local neuropil-based correction was similar to what previously done in raster scanning (Chen et al., 2013), and it was effective in decreasing correlation across ROIs. The global neuropil and the corrected PC1 of all ROIs can also be effective in decreasing correlation across ROIs, and they have the advantage that they can be computed even when the surround region is zero.

### Population Imaging with Single AP Resolution Using SLS

**Figure 5:**
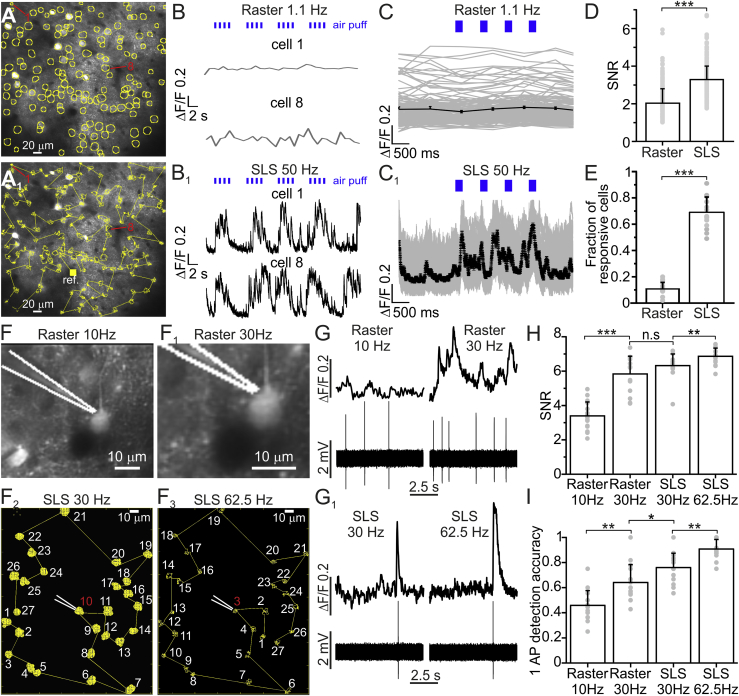
SLS Allows Single AP Resolution during Population Imaging (A) GCaMP6s-expressing layer IV principal neurons. Yellow circles identify 155 neurons. (A_1_) SLS trajectory (yellow line) crossing all the identified cells. (B and B_1_) Fluorescence over time from the two cells indicated in red in (A) in conventional raster scanning acquisitions (B) and SLS (B_1_). Blue ticks above traces indicate whisker stimuli (duration: 200 ms). (C and C_1_) Fluorescence over time of all cells shown in (A) (gray traces) during consecutive whisker stimulations and the average across cells (black trace) in raster scanning (C) and SLS (C_1_). (D) SNR of events in all raster scan series and all SLS acquisitions. n = 360 cells from 4 animals; paired t test, p = 5E-4. (E) Fraction of cells responding to the whisker stimulation in raster scanning and SLS. n = 360 cells from 4 animals; paired t test, p = 4E-12. (F–F_3_) Simultaneous juxtasomal recording and raster scan experiment from a GCaMP6s-expressing neuron at 10 Hz (F) and 30 Hz (F_1_). The white line indicates the recording pipette. The same cell in (F) is recorded using juxtasomal recording and SLS imaging in a much larger FOV at 30 Hz (F_2_) and 62.5 Hz (F_3_). The yellow line in (F_2_) and (F_3_) represents the SLS trajectory across the different cells (white number). The same cells were differently numbered in SLS at 30 Hz and 62.5 Hz because the SLS trajectory changed when the surround region was modified to change the scanning rate. (G) Left: fluorescence over time acquired in raster scanning at 10 Hz (top) and electrophysiology recording (bottom). Right: fluorescence over time acquired in raster scanning at 30 Hz (top) and electrophysiology recording (bottom). (G_1_) The same as in (G) for SLS at 30 Hz (left) and at 62.5 Hz (right). (H) SNR of fluorescence events associated to isolated APs (see STAR Methods) under the different conditions. n = 16 acquisitions from 8 FOVs in 4 animals; Wilcoxon rank-sum test; p = 5E-4 for raster scan at 10 Hz versus raster scan at 30 Hz; p = 0.12 for raster scan at 30 Hz versus SLS at 30 Hz; p = 7E-3 for SLS at 30 Hz versus SLS at 62.5 Hz. (I) Accuracy of single AP detection under the different conditions. n = 16 acquisitions from 8 FOVs in 4 animals; Wilcoxon rank-sum test; p = 2E-3 for raster scan at 10 Hz versus raster scanning at 30 Hz; p = 0.01 for raster scan at 30 Hz versus SLS at 30 Hz; p = 2E-3 for SLS at 30 Hz versus SLS at 62.5 Hz.

We performed population calcium imaging in large FOVs containing hundreds of cells in the barrel cortex of anesthetized mice using the SLS and conventional raster scanning (Figures 5A and 5A1). The same set of cells (360 in N = 4 animals) was imaged under the different conditions (acquisition rate: 49 ± 2 Hz for SLS, 1.0 ± 0.1 Hz for raster scanning; same dwell time (4.4 μs) and excitation power (30–90 mW) for both conditions). Neuronal activity was stimulated by repetitive deflections of the whiskers with an air puff. In raster scanning, small fluorescence events were associated to the sensory stimulus (gray in Figures 5B and 5C), whereas in SLS cells displayed clear calcium transients in response to the whisker deflection (black in Figure 5B1, gray in Figure 5C1). The SNR of fluorescence transients (Figure 5D) and the fraction of responsive neurons (Figure 5E) were higher for SLS than for raster scanning.

To compare the accuracy in detecting APs over the population of cells in SLS with that obtained with raster scanning, we performed simultaneous juxtasomal electrophysiological and imaging recordings (Figures 5F–5I). Imaging was performed at 30–62.5 Hz in the SLS approach over tens of different cells. Raster scanning was performed on small FOVs, including the cell body of the recorded neurons and a small surrounding region at 10–30 Hz. We found that the SNR of fluorescence events recorded with SLS at 30 Hz had a similar SNR of fluorescence transients recorded in the raster scanning at the same acquisition frequency of 30 Hz (Figures 5G and 5H). Raster scanning at 10 Hz reduced the SNR associated with fluorescence transients compared to 30 Hz. In contrast, SLS at 62.5 Hz increased the SNR of fluorescence signals compared to that of events recorded at 30 Hz (Figure 5H). The accuracy in detecting a single AP was an increasing function of the acquisition rate both in SLS and in raster scanning (Figure 5I). Importantly, the accuracy in detecting a single AP in SLS over a large group of cells was higher than that recorded in raster scanning (Figure 5I), and it reached the value of 0.9 at 62.5 Hz, close to that previously reported for single cells in raster scanning at similar acquisition frame rate (Chen et al., 2013), but scanned a much smaller FOV including one or few cells. Altogether, these results demonstrate that SLS allows us to obtain, at the population level, the highest performance in detecting AP that raster scanning achieves at the single-cell level, thus enabling high-accuracy population imaging.

We investigated how the SNR and the accuracy in detecting AP from fluorescence signals depended on the acquisition rate and on the dwell time per cell. To this aim, we temporally or spatially downsampled SLS and raster scanning acquisitions. We found that downsampling lines (for SLS) or frames (for raster scanning) in time led to a mild but significant decrease in the SNR and in the accuracy in detecting APs (Figures S7A–S7D1). In SLS, downsampling the acquisition rate to half reduced the SNR by ∼7% and the accuracy by ∼6% (Figures S7C and S7D). In raster scanning, downsampling to one-third data from series acquired at 30 Hz led to SNR and accuracy values higher than those obtained by raster scanning a larger FOV containing the same cell at 10 Hz (Figures S7C1 and S7D1). We observed a stronger dependence of the SNR and accuracy on the number of pixels per cell (which is proportional to the dwell time per cell). In SLS, decreasing the number of pixels to half reduced the SNR by ∼31% and the accuracy by ∼33% (Figures S7G and S7H). In raster scanning mode, decreasing the number of pixels to half reduced the SNR by ∼22% and the accuracy by ∼15% (Figures S7G1 and S7H1). In summary, these results show that both the SNR and the accuracy in detecting single APs depended on both the acquisition rate and the dwell time per cell. However, these findings also suggest a stronger dependence of SNR and accuracy on the dwell time per cell rather than on the acquisition rate, which is in line with the relatively slow kinetics of the GECI GCaMP6s.

We controlled for potential photodamage introduced by the SLS modality by performing one SLS acquisition, on average, every 420 s for a total of 4 h. We found that the firing rate of imaged cells recorded in juxtasomal configuration did not change between the first and the 4th h of imaging sessions (average firing rate estimated in 120 s: 0.29 ± 0.13 Hz during the first hour versus 0.29 ± 0.08 Hz during the 4th h of the imaging session; paired t test, p = 0.85 from 4 FOVs in 4 anesthetized animals). Moreover, the number of total GCaMP6s-expressing cells and number of cells with a filled fluorescence in the FOVs remained constant between the 1st and the 4th h of the imaging session (total number of identified cells within the 1st h: 83 ± 28; total number of identified cells within the 4th h: 83 ± 28; number of filled cells within the 1st h: 2 ± 1; number of filled cells within the 4th h: 2 ± 1; data from 4 FOVs in 4 anesthetized mice; paired t test between distribution of total detected cells and filled cells during 1st versus 4th hour, p = 1).

### High-Accuracy Mapping of Neural Ensembles in Awake Mice

**Figure 6:**
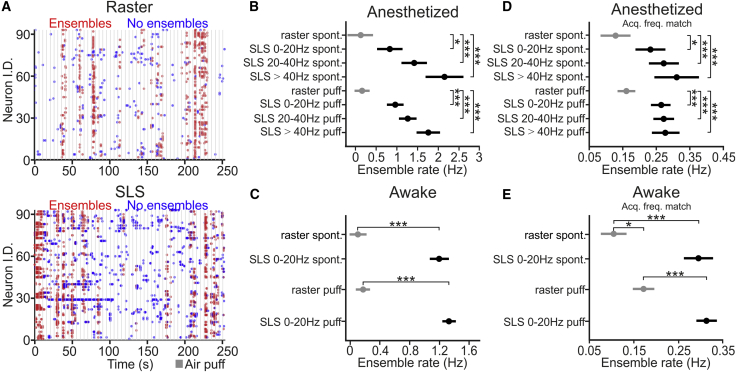
Increased Rate of Detected Neural Ensembles in SLS (A) Top: functional ensembles (red dots) detected in an awake animal using raster scan imaging (frame rate: 0.8 Hz). Bottom: functional ensembles (red dots) detected in the experiment shown in the top panel using SLS (frame rate: 48 Hz). The gray vertical shades indicate whisker stimuli. Blue dots indicate active neurons not belonging to any ensemble. (B and C) Rate of detected ensembles in anesthetized (B) and awake (C) animals under the different conditions. Data are classified according to modality (raster or SLS at different acquisition rates) and to the type of activity (spontaneous or air puff stimulation). Spontaneous activity in (B): n = 6 for raster, n = 6 for SLS at 0–20 Hz, n = 6 for SLS at 20–40 Hz, n = 3 for SLS at >40 Hz from 6 anesthetized mice. Paired t test, p = 0.01 for raster versus SLS 0–20 Hz, p = 3E-7 for raster versus SLS 20–40 Hz; unpaired t test, p = 6E-8 for raster versus SLS >40 Hz. Air puff stimulation in (B): n = 20 for raster, n = 15 for SLS at 0–20 Hz, n = 13 for SLS at 20–40 Hz, n = 7 for SLS at >40 Hz from 6 anesthetized mice. Unpaired t test, p = 1E-7 for raster versus SLS 0–20 Hz, p = 6E-8 for raster versus SLS 20–40 Hz, p = 6E-7 for raster versus SLS >40 Hz. Spontaneous activity in (C): n = 5 for raster and n = 4 for SLS at 0–20 Hz from 2 awake mice; unpaired t test, p = 4E-9. Air puff stimulation in (C): n = 7 for raster and n = 8 for SLS at 0–20 Hz from 2 awake mice; unpaired t test, p = 4E-9. (D and E) Same as in (B) and (C) but for SLS downsampled to match the acquisition frequency of raster scanning (Acq. freq. match). Spontaneous activity in (D): paired t test, p = 0.01 for raster versus SLS 0–20 Hz, p = 1E-4 for raster versus SLS 20–40 Hz; unpaired t test, p = 5E-5, for raster versus SLS >40 Hz. Air puff in (D): unpaired t test, p = 2E-6 for raster versus SLS 0–20 Hz, p = 7E-7 for raster versus SLS 20–40 Hz, p = 3E-5 for raster versus SLS >40 Hz. Spontaneous activity in (E): unpaired t test, p = 2E-7. Air puff stimulation in (E): t test, p = 3E-7. Unpaired t test, p = 0.01, for raster in spontaneous versus raster in air puff stimulation.

**Figure 7:**
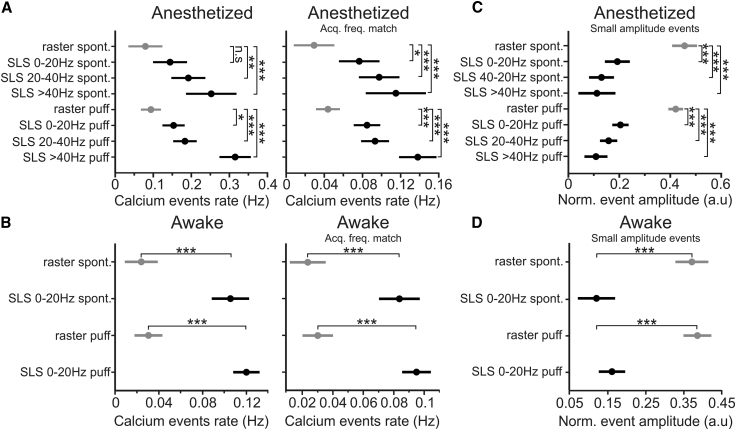
Increased Rate of Detected Calcium Events in SLS (A) Left: rate of detected calcium events in anesthetized animals under the different conditions. Right: rate of detected events with downsampling of SLS to match the acquisition rate of raster scanning. Spontaneous activity: n = 6 for raster, n = 6 for SLS at 0–20 Hz, n = 6 for SLS at 20–40 Hz, n = 3 for SLS at >40 Hz from 6 anesthetized mice. Left: paired t test, p = 0.32 for raster versus SLS 0–20 Hz, p = 4E-3 for raster versus SLS 20-40 Hz; unpaired t test, p = 1E-4 for raster versus SLS >40 Hz. Right: paired t test, p = 0.02 for raster versus SLS 0–20 Hz, p = 1E-4 for raster versus SLS 20–40 Hz; unpaired t test p = 1E-4 for raster versus SLS >40 Hz. Air puff stimulation: n = 20 for raster, n = 15 for SLS at 0–20 Hz, n = 13 for SLS at 20–40 Hz, n = 7 for SLS at >40 Hz from 6 anesthetized mice. Left: unpaired t test, p = 0.01 for raster versus SLS 0–20 Hz, p = 1E-4 for raster versus SLS 20–40 Hz, p = 1E-4 for raster versus SLS >40 Hz. Right: unpaired t test, p = 1E-4 for raster versus SLS 0–20 Hz, p = 3E-6 for raster versus SLS 20–40 Hz, p = 6E-8 for raster versus SLS >40 Hz. (B) Same as in (A) for awake mice. Spontaneous activity: n = 5 for raster and n = 4 for SLS at 0–20 Hz from 2 awake mice. Unpaired t test, p = 4E-6 and p = 9E-6 for left and right panels. Air puff stimulation: n = 7 for raster and n = 8 for SLS at 0–20 Hz from 2 awake mice. Unpaired t test, p = 2E-8 and p = 6E-8 for left and right panels. (C) Normalized amplitude of events under the different conditions in anesthetized mice. Same dataset as in (A). Spontaneous activity: paired t test, p = 6E-8 for raster versus SLS 0–20 Hz, p = 6E-8 for raster versus SLS 20–40 Hz; unpaired t test, p = 6E-8 for raster versus SLS >40 Hz. Air puff stimulation: unpaired t test, p = 6E-8 for raster versus SLS 0–20 Hz, p = 6E-8 for raster versus SLS 20–40 Hz, p = 6E-8 for raster versus SLS >40 Hz. (D) Same as in (C) for awake mice. Number of acquisitions for each condition as in (B). Spontaneous activity: unpaired t test, p = 2E-6. Air puff stimulation: unpaired t test, p = 1E-7.

We used SLS to characterize population dynamics in anesthetized and awake animals during spontaneous and sensory-evoked activities. We first aimed for the identification of neuronal ensembles comparing results between imaging sessions acquired using raster scan and SLS. SLS traces were classified according to acquisition rate in 3 classes: 0–20 Hz, 20–40 Hz, and >40 Hz. Ensemble detection was performed as described in Miller et al. (2014) (see STAR Methods). We found that functional ensembles were more frequently observed in SLS acquisitions than in raster scanning (Figure 6A). Indeed, ensemble rate was higher for SLS than for raster scanning during spontaneous and sensory-evoked activities in both anesthetized (Figure 6B) and awake mice (Figure 6C). The increased ensemble rate observed in SLS could be due to the increased acquisition rate in this modality than in raster scanning. To control for this, we downsampled SLS acquisitions to match the acquisition rate of raster scanning. We found that the ensemble rate of spontaneous and sensory-evoked activities was still higher in downsampled SLS than in raster scanning in both anesthetized (Figure 6D) and awake mice (Figure 6E), suggesting that the increased rate of ensembles in SLS might be due to increased accuracy in detecting small calcium events. In agreement with this, we observed that the rate of detected calcium events for spontaneous and sensory-evoked activities was higher for SLS than for raster scanning in both anesthetized (Figure 7A, left) and awake mice (Figure 7B, left). The higher calcium event rate in SLS was still observed when SLS acquisitions were downsampled to match the acquisition frequency of raster scanning (Figures 7A and 7B, right). The coactivity threshold (left panels in Figures S8A and S8B) was lower and the time to peak of calcium events (Figures S8C and S8D) was smaller in SLS than in raster scanning. Moreover, we defined “small” calcium events as those events with amplitude below a certain threshold compared to local maxima in the fluorescence trace (Figure S9A; see also STAR Methods for details). The amplitude of these small events was smaller in SLS than in raster scanning (Figures 7C and 7D; Figure S9B).

We used a non-negative matrix factorization (NMF) to decompose the spatial pattern of calcium activity observed across ROIs at each instant of time where an ensemble was detected into the sum of a given number of activity patterns that mostly recurred during the recording (called modules) with non-negative activation coefficients. We calculated the percentage of variance accounted for (VAF) of calcium signals by using a spatial NMF decomposition of imaging data when increasing the number of spatial modules used to decompose neural activity (see STAR Methods). To quantify the diversity of spatial patterns of activity expressed at each instant of time, we quantified how the VAF depended on the number of spatial modules used in the NMF decomposition (the number was normalized to the maximal number of possible modules, which was equal to the number of ROIs). The more modules were needed to reach a given percentage of VAF, the more diverse were the patterns expressed by neural activity. We found that higher numbers of modules were needed to reach a given VAF level for SLS than for raster scanning (Figures S9D and S9E), suggesting that SLS picked a more diverse range of instantaneous spatial activation patterns than the raster scan did. Upon downsampling of SLS (SLS binned in Figures S9D and S9E), this difference in VAF was no longer observed, suggesting that this was due to the higher acquisition rate obtained in SLS. Moreover, the spatial sparseness of functional modules (computed in each FOV using the number of modules giving 50% of VAF) was higher in SLS than in raster scanning (Figure S9F), suggesting that instantaneous firing patterns captured by SLS were more spatially concentrated than those captured by the raster scans. Taken together, the results of more diverse and more spatially localized instantaneous firing patterns suggest that SLS separates into finer instantaneous spatial patterns than what would have been artificially collated into a single, less spatially localized instantaneous activation pattern by the raster scanning. These findings are consistent with an increased precision of population imaging by using SLS compared with raster scanning as a combined effect of both increased scanning speed and higher SNR. Indeed, although temporal downsampling of SLS decreased the VAF to raster scan levels (Figures S9D and S9E), ensemble rate remained higher (Figures 6D and 6E).

If SLS allowed a more precise characterization of ensemble activity than raster scanning, more accurate decoding of the external state (air puff presence/absence) from SLS trajectories should be expected. To test this hypothesis, we trained a support vector machine (SVM) (Bzdok et al., 2018) on 50% of data and tested classification performance on the remaining 50% of data. We found that SVM decoded the presence/absence of the sensory stimulus with higher accuracy in SLS acquisitions than raster scanning (see STAR Methods; accuracy above chance level for SLS: 0.09 ± 0.02, n = 30; accuracy above chance level for raster scan: 0.01 ± 0.03, n = 20; Wilcoxon rank-sum test, p = 3E-8).

## Discussion

We developed a method, SLS, to increase the SNR in population GCaMP6 imaging. SLS combines image segmentation using pixel-wise statistics and line scan trajectories, and it samples only the pixels with the most information within each cell. SLS allowed population imaging with single AP resolution, and it increased the frequency of detected neuronal ensembles compared to raster scanning. The observed increase in ensemble frequency was paired with increased frequency of detected calcium events and decreased average amplitude of small calcium events. This suggests that SLS allows a richer description of neural ensembles because of increased accuracy in detecting small events associated with low firing rates or low GCaMP6 expression levels. In agreement with this conclusion, (1) the coactivity threshold was smaller in SLS than in raster scanning, (2) a larger number of modules in the NMF decomposition had smaller VAF in SLS than in raster scanning, and (3) the sparseness of modules explaining 50% of variance was larger for SLS than for raster scanning.

These results are of importance to identify the circuit mechanisms underlying behavior because they allow better correlation of activity patterns with behavioral variables. In line with this, the improved accuracy in detecting ensemble activity was associated with better performance of SVM in decoding the presence/absence of the sensory stimulus. These findings are also relevant for all optical imaging and holographic manipulation approaches, where precise identification of neural ensembles is the first crucial step to guide subsequent optogenetic manipulations (Forli et al., 2018, Packer et al., 2015, Panzeri et al., 2017).

Different line scan methods have been developed to increase frame rate by reducing the number of scanned pixels. These methods have been so far applied *in vitro* and in anesthetized animals (Kim et al., 2012, Langer et al., 2013, Lillis et al., 2008, Sadovsky et al., 2011, Schuck et al., 2018, Valmianski et al., 2010). However, previous line scan approaches have not been extensively applied in awake animals and to GECI, the most commonly used class of functional indicators nowadays. Moreover, previous work did not extensively address the issue of neuropil decontamination nor that of dealing with motion artifacts. Building on those important studies, the SLS method that we described here is characterized by important additional implementations. First, SLS is generated to maximize SNR from each cell in a pixel-parsimonious way (on average 25% of the cell’s pixels are scanned in SLS), allowing for signal quality improvement and fast acquisition rates (30–120 Hz for hundreds of cells). Second, we applied SLS to image GCaMP6s, achieving high accuracy in detecting AP in population imaging. This allowed better detection of small calcium signals associated with the discharge of isolated APs at the level of neuronal population, a result obtained so far imaging only individual or few cells (Chen et al., 2013). Third, we implemented background subtraction in SLS by using different strategies. Fourth, we devised a strategy to identify large movement artifacts, and we implemented a method to try to compensate for small movements. In raster scanning imaging, movement artifacts are usually corrected for by using various methods, which rely on the anatomical features contained in the image (Dubbs et al., 2016, Pnevmatikakis and Giovannucci, 2017). In SLS, compensation for motion distortions during acquisition was achieved incorporating a surround region for each ROI and then relying on SNR-based pixel reassignment in a sliding window of 10 s (the sliding window was moved in the temporal axis by one frame at a time).

The pixel reassignment procedure did not allow frame-by-frame correction for motion artifacts. Moreover, the autoregressive procedure allowed the identification of large motion artifacts but required us to discard data after the detection of one such event. To achieve frame-by-frame correction of motion artifacts, an approach similar to Nadella et al. (2016) and Szalay et al. (2016) could be developed. Additionally and in a more complex experimental design, the movement-induced ROI shift in the X and Y directions for each ROI could be recovered by imaging a second static red channel by using raster scanning with an independent scan head. Alternatively and assuming rigid movement of the whole FOV during motion artifacts, the X and Y shifts for ROIs could be recovered from the reference patch.

SLS could also be achieved steering the beam with acousto-optic deflectors (AODs) (Akemann et al., 2015, Duemani Reddy et al., 2008, Grewe et al., 2010, Nadella et al., 2016, Otsu et al., 2008) rather than galvanometric mirrors. This would have the advantage of removing the correlation between the time needed to steer the beam from one position to another and the physical distance between the two beam positions. However, AODs introduce significant limitations, such as spatial distortions of the beam, significant increase in the group dispersion velocity, relatively small FOV, and high costs. In contrast, the approach described in this study reduces costs and it is applicable to most galvanometric mirror-based scopes.

Optimization of the fluorescence excitation based on pixel-wise SNR may increase the SNR and reduce photodamage in functional voltage and calcium imaging using holographically sculpted light schemes (Bovetti et al., 2017, Castanares et al., 2016, Dal Maschio et al., 2010, Foust et al., 2015, Nikolenko et al., 2008, Szabo et al., 2014, Tanese et al., 2017, Yang et al., 2015). SLS is characterized by high SNR and high acquisition rates. Thus, future applications of this technology may facilitate time-resolved lifetime fluorescence (Zheng et al., 2018) and voltage imaging (Chamberland et al., 2017, Xu et al., 2017). However, when implementing SLS to image indicators with faster kinetics than GCaMP6, the tradeoff between acquisition rate and dwell time per cell will probably need to be reconsidered. Resonant raster imaging may achieve frame rates of 30 Hz that are comparable with the acquisition rates of some of the SLS recordings presented in this study. However, SLS can achieve higher frame rates and it is characterized by an inherently higher ratio between the cellular dwell time and total frame time than imaging with resonant mirrors. For this reason, SLS may be particularly useful in the presence of sparse cellular labeling such that observed in some transgenic animal models expressing GECIs (Chen et al., 2012, Song et al., 2017) and when imaging specific classes of interneurons (Ebina et al., 2014, Kato et al., 2013) or small astrocyte structures displaying fast calcium kinetics (Bindocci et al., 2017, Stobart et al., 2018). Future developments of SLS may include 3D SLS using electrically tunable lenses (Grewe et al., 2011), two-color SLS, and SLS of functional indicators combined with holographic optogenetic stimulation (Bovetti and Fellin, 2015, Forli et al., 2018, Mardinly et al., 2018, Packer et al., 2015, Papagiakoumou et al., 2010, Yang et al., 2018).

In conclusion, we developed a method for population imaging of GCaMP6 with single AP resolution in anesthetized and awake head-fixed rodents. This technique is easily implementable on commercial scanning two-photon microscopes, allowing precise identification of neural ensembles during population imaging *in vivo* with no change in the microscope optical pathway.

## STAR★Methods

### Key Resources Table

**Table:** 

REAGENT or RESOURCE	SOURCE	IDENTIFIER
**Bacterial and Virus Strains**		

AAV.Syn.Flex.GCaMP6s.WPRE.SV40	Penn Vector Core Chen et al., 2013	RRID: Addgene_100845;
		Addgene viral prep # 100845-AAV1

**Chemicals, Peptides, and Recombinant Proteins**		

Alexa Fluor 488 hydrazide, sodium salt	Thermo Fisher Scientific	Cat# A10436

**Experimental Models: Organisms/Strains**		

Mouse: B6;C3-Tg(Scnn1a-cre)3Aibs/J	The Jackson Laboratory	RRID:IMSR_JAX:009613
Mouse: B6;129S6-*Gt(ROSA)26Sor*^*tm14(CAG-TdTomato)Hze*^/J	The Jackson Laboratory	RRID:IMSR_JAX:007908

**Software and Algorithms**		

MATLAB R2017b	Mathworks	RRID:SCR_001622; URL: https://www.mathworks.com/products/matlab/
Origin(Pro) 2018 64bit	OriginLab	RRID:SCR_014212; URL: http://www.originlab.com/index.aspx?go=PRODUCTS/Origin
ImageJ (version 1.52e)	Fiji, Schindelin et al., 2012	RRID:SCR_002285; URL: http://fiji.sc
NoRMCorre	Pnevmatikakis and Giovannucci, 2017	URL: https://github.com/flatironinstitute/NoRMCorre
CaImAn	Giovannucci et al., 2019	URL: https://github.com/flatironinstitute/CaImAn-MATLAB
FluoroSNNAP	Patel et al., 2015	URL: https://www.seas.upenn.edu/∼molneuro/software.html
Traveling Salesman Problem - Genetic Algorithm version 1.3.0.0 (9.62 KB) by Joseph Kirk	MATLAB Central File Exchange 2017	URL: https://www.mathworks.com/matlabcentral/fileexchange/13680-traveling-salesman-problem-genetic-algorithm
HOPS (Heuristically optimal path scanning)	Sadovsky et al., 2011	URL: http://macleanlab.uchicago.edu/software/
pClamp 10 (version 10.4) Software Suite	Molecular Devices	RRID:SCR_011323URL: https://www.moleculardevices.com

**Other**		

Kwik-Cast	World Precision Instruments	Cat# KWIK-CAST
MultiClamp 700B amplifier	Molecular Devices	URL: https://www.moleculardevices.com
Axon Digidata 1440A	Molecular Devices	URL: https://www.moleculardevices.com

### Lead Contact and Materials Availability

Further information and requests for resources and reagents should be directed to and will be fulfilled by the Lead Contact, Tommaso Fellin (tommaso.fellin@iit.it). This study did not generate new unique reagents.

### Experimental Model and Subject Details

#### Mice

All experiments were carried out according to the guidelines of the European Communities Council Directive and approved by the Istituto Italiano di Tecnologia (IIT) Animal Health Regulatory Committee and by the National Council on Animal Care of the Italian Ministry of Health (authorization #34/2015-PR). Animals used in this study were obtained by crossing B6;C3-Tg(Scnn1a-cre)3Aibs/J (JAX #009613, called Scnn1a-cre line) with C57BL/6J mice (Charles River, Calco, Italy), or, for Figure S1, with B6;129S6-*Gt(ROSA)26Sor*^*tm14(CAG-TdTomato)Hze*^/J (JAX #007908, tdTomato line). All transgenic strains were purchased from the Jackson Laboratory (Bar Harbor, USA). Animals were housed in individually ventilated cages under a 12-hr light:dark cycle. A maximum of five animals *per* cage was allowed. Access to food and water was *ad libitum*. Data included in the present work come from a total of 16 animals (both sexes).

### Method Details

#### Viral injections

AAV1.Syn.Flex.GCaMP6s.WPRE.SV40 was purchased from the University of Pennsylvania Viral Vector Core. Viral injections were performed on postnatal days 30-33 (P30–P33). Animals were anesthetized with 2% isoflurane/0.8% oxygen, placed into a stereotaxic apparatus (Stoelting Co, Wood Dale, IL), and maintained on a warm platform at 37°C for the whole duration of the anesthesia. After scalp incision, two small holes were drilled on the skull above the somatosensory cortex at stereotaxic coordinates [-1.2, −2] mm from bregma in the antero-posterior direction and [2.8, 3] mm in the lateral direction to lower the micropipette into the tissue (pipette depth: 0.3 - 0.4 mm from the pia) at the two sites. 200 nL of virus were injected at 20 - 60 nl/min at each site by means of a hydraulic injection apparatus driven by a syringe pump (UltraMicroPump, WPI, Sarasota, FL). The injected solution contained viral particles in 10^12^ genomes/ml diluted 1:1 in artificial cerebro-spinal fluid (aCSF). At the end of the procedures, the skull was cleaned and the skin incision sutured and cleansed with betadine. Animals were monitored until full recovery and experiments performed 10-20 days after the injection.

In mice used for imaging in awake conditions (see below), a custom metal plaque was sealed on the skull to assurance stable head fixation during two-photon imaging using vetbond (3 M, St. Paul, MN, USA) and dental cement (Paladur, Kulzer GmbH). The exposed bone was covered using the silicone elastomer KWIK-Cast (World Precision Instruments, Friedberg, DE) and an intraperitoneal injection of antibiotic (BAYTRIL, Bayer, DE) was performed.

#### Animal preparation for two-photon imaging

For imaging in anesthetized animals, mice were injected intraperitoneally with urethane (16.5%, 1.65 g/kg). The scalp was cut after infiltrating all incisions with lidocaine (2%). Using an epifluorescence stereoscope, the position of brighter fluorescence emission within the somatosensory cortex was identified and a custom made plastic chamber with a central hole (hole diameter: 4 mm) positioned on top of the high fluorescence region was attached with dental cement to the animal’s skull for head-fixation (Bovetti et al., 2017). A craniotomy was opened over the targeted cortical area while leaving the meninges intact. The craniotomy was round and ∼0.5 mm in diameter for imaging experiments and rectangular (0.5 mm x 2 mm) for combined imaging and juxtasomal recordings (see below). The surface of the brain was kept moist with normal HEPES-buffered aCSF composed of: 127 mM NaCl, 3.2 mM KCl, 2 mM CaCl_2_, 1 mM MgCl_2_ and 10 mM HEPES [pH 7.4] at 37°C. Body temperature was maintained at 35°C with a heating pad. Respiration rate, heartbeat, eyelid reflex, vibrissae movements, and reactions to tail pinching were typically monitored throughout the surgery. Once the craniotomy was performed, animals were moved under the two-photon microscope, the body temperature kept at 37°C, and the brain surface irrigated with aCSF maintained at 35-36°C. Imaging session began one hour after the end of the surgeries procedures.

For experiments in awake animals, mice were trained for a period of 7–10 days to head fixation before two-photon imaging was performed. During this training period, animals were head restrained for increasing temporal windows (from 15 minutes to one hour) and free to run on a wheel. On the day of the experiment, the habituated animal was anesthetized with isoflurane (2%) to perform a craniotomy with procedures similar to the ones described above. After surgery, the animal was head fixed and allowed to recover under the microscope for at least one hour before imaging.

#### Raster scanning and SLS two-photon imaging

Two-photon GCaMP6s imaging was performed using a chameleon ultra II pulsed laser (80 MHz pulse frequency, Coherent, Milan, IT) tuned at 920 nm. The range of excitation power under the objective was assessed with a power meter and typically set between 30 mW and 90 mW for layer IV imaging. An Ultima II scanhead (Bruker Corporation, Milan, IT) and an Olympus 25X 1.05 N.A. (XLPLN25XWMP2, Olympus, Milan, IT) were used for two-photon imaging. The scanhead (Bruker Corporation, Milan, IT) was equipped with a pair of galvanometric mirrors (open aperture 3 mm, 6215H, Cambridge Technology, USA). The driver board of the galvanometric mirrors was customized (named 6SPRAIRIE5, Cambridge Technology, USA) and it was calibrated in-house by the scanhead producer (Bruker Corporation, Milan, IT) for fast settling with a 1 V step response. No high power option was used. For raster scanning and line scan imaging, the dwell time was fixed at 4.4 μs, the photomultiplier voltage to 777 V, the zoom factor to 1 for all experiments. The FOV surface was adjusted in each experiment according to the number and position of GCaMP6 expressing cells. Pixel size 0.77 μm. Excitation power was adjusted to obtain pixel values across 95% of the available dynamic range (16 bit).

#### Semi-automatic FOV segmentation and generation of SLS trajectories

To generate SLS trajectories, a reference raster scanning t-series was first acquired (60-100 frames). Manual identification of the center of individual cells was performed on the average temporal projection of the t-series or frame by frame (Figure 2). A square box was then generated and centered on the selected positions. The box dimensions were visually adjusted to best fit the cell perimeter. The average lateral dimension of the box (11.8 ± 0.7 μm, Figure 2) was not observed to vary considerably across cells in a given t-series. The dimension of the first box in a t-series was then typically used to fit the vast majority of visible cells, speeding up the segmentation process. Upon selection of one box, the software identified the frame displaying the highest fluorescence value in that box across frames of the t-series. At this point, pixels inside each box were ranked according to their SNR (from high SNR to low SNR) and the average SNR across selected pixels was plotted as a function of number of selected pixels. The SNR of the pixel i,j, SNR_i,j_, was defined as:

$$SNR_{i,\;j}=\frac{\max_{{\;}_{\;}^{\text{t}\in \left[\text{t}1,\text{t}2\right]}}F_{i,\;j}\left(t\right)}{noise_{i,\;j}}$$

where *max F*_*i,j*_*(t)* is the maximum fluorescence intensity of the pixel i,j over the considered time interval Δt = t_2_-t_1_, 1 < *i* < N_x_, 1 < *j* < N_y_, N_x_ and N_y_ are the number of image pixels in the x and y direction, and *noise*_*i,j*_ was computed as the standard deviation across all fluorescence values below the 25^th^ percentile of the fluorescence distribution of the pixel *i,j* in the same time interval Δt. The SNR of the ROI was computed as follows:

SNR=maxm∈[[1,M]⟨Fm(t)⟩noise

where *max < F*_*m*_*(t) >* is the maximum of the average intensity of individual pixels over time, < F_m_(t) > , for the M pixels belonging to a given ROI in the time interval Δt (with t_1_ < t < t_2_ and 1 < *m* < M). *noise* was computed as the standard deviation across all fluorescence values below the 25^th^ percentile of the fluorescence distribution of the M different pixels belonging to the ROI in the time interval Δt.

These plots typically showed a peak followed by a plateau or a decrease (Figure 2E). The software automatically identified the maximum of the SNR plot for one box (this value could be adjusted by the user if needed). All pixels with SNR higher than or equal to the maximum of the average SNR plot were chosen as representative for the cell corresponding to the considered box. We observed that the number of pixels used to maximize the SNR in one cell was different across cells and it typically ranged between 30 and 50. Once iterated across boxes, this procedure returned a set of pixels for each cell with no overlap across pixels belonging to different cells. Neuropil and nuclear regions were typically excluded because of their low SNR. When all visible cells were identified, a genetic algorithm (https://www.mathworks.com/matlabcentral/fileexchange/13680-traveling-salesman-problem-genetic-algorithm) was used to determine the optimal path for the SLS. Each selected pixel was scanned by the SLS trajectory and the sequence of cells was selected to produce the shortest trajectory. The first and the last pixels of the SLS trajectory were chosen from two adjacent cells in order to generate a closed path and reduce fly-back time (Sadovsky et al., 2011) (http://macleanlab.uchicago.edu/software/). A surround region was then added to the trajectory. The surround region covered the area around the pixels which were previously found to maximize the SNR in the ROIs (ROI pixels). The dimension of the surround area could be adjusted and it was determined as including those pixels with distance 0, 1, 2, 3 pixels from the ROI pixels (with the value 0, 1, 2, 3 selected by the user). All the SLS trajectories were saved as .xml files, a format compatible with the image acquisition system. Computation time for the generation of line scan trajectories was < 10 s. The number of line scan trajectories was directly controlled in the image acquisition software. When comparing between raster and SLS all acquisition parameters (pixel dwell time, pixel size, laser power, etc.) were kept constant. No visible sign of photodamage or photobleaching was observed during prolonged SLS.

When test trajectories were used to measure the temporal lag between the command signal and feedback signals as a function of the angle between successive segments of the line trajectory, each test trajectory was composed of regular polygons with a lateral size of 20 pixels. The angles between segments increased across trajectories in step of 20° in the range [20°, 160°] and polygons were repeated several times along the horizontal side of the FOV. Individual polygons were separated by horizontal segments.

#### Combined two-photon imaging and juxtasomal electrophysiological recording

Temporal series from GCaMP6s expressing neurons were acquired in the raster scanning configuration at 10 Hz or 30 Hz and in the SLS at 30 Hz or 62.5 Hz while simultaneously recording the spiking activity of one of the imaged neurons with a patch pipette. To collect imaging sessions at the specific acquisition rates listed above, the dimensions of the field of view (for raster scanning) and of the surround regions (for SLS) were adjusted accordingly. Two-photon targeted juxtasomal electrophysiological recordings were performed as previously described (De Stasi et al., 2016, Zucca et al., 2017). Borosilicate glass pipettes had resistance of 7 - 12 MΩ (tip diameter: 1 - 1.2 μm) and were filled with aCSF mixed with Alexa Fluor 488 (20 mM) for pipette visualization. Neurons were identified and targeted based on the signal generated by the cellular fluorescent reporter (GCaMP) using two-photon excitation at λ = 920 nm. Glass pipettes were inserted with a shallow angle (∼25°) in the brain tissue and slowly advanced toward the targeted neuron. Once the pipette reached the proximity of the target cell, gentle suction was applied until the resistance between the cell membrane and the glass reached a value between 50 MΩ and 300 MΩ. Electrophysiological signals were amplified and filtered at 8 kHz by a MultiClamp 700B amplifier (Molecular Devices, Sunnyvale, CA) and digitized at 10 kHz using a Digidata 1440A (Molecular Devices, Sunnyvale, CA).

#### Whisker stimulation

A picospritzer (Intracel, Royston Herts, UK) was used to deliver a train of 4 air puffs (duration, 200 ms; pressure, 1.5 psi) with duty cycle 0.5. Each stimulation trial consisted of 4 trains at 0.5 Hz. The picospritzer output was relayed with tygon tubing to a syringe needle placed in proximity of whisker field contralateral to the imaging site.

#### Image analysis

In order to classify fields of view according to the GCaMP6s expression levels across animals, we measured the average fluorescence in the whole FOV and across the whole t-series from reference t-series acquired in raster scan mode from different animals (N = 8 t-series from 8 anesthetized animals). FOVs with the 4 largest average values of fluorescence were classified as belonging to high expressing animals, while the remaining 4 as belonging to low expressing animals (average absolute fluorescence intensity from 4 high expressing animals: 2,657 ± 605 bit; average absolute fluorescence intensity from 4 low expressing animals: 1,526 *±* 181 bit, Wilcoxon rank-sum test p = 0.012). The percentage of filled cells with high expression level was not different in high and low expressing FOVs (0.5 ± 0.3% N = 4 in high expressing FOVs and 0.6 ± 0.3% N = 4 in low expressing FOVs, Wilcoxon rank-sum test p = 0.5).

#### Processing of GCamp6 t-series

##### Motion correction and neuropil decontamination

For t-series acquired with conventional raster scan, we first corrected for motion artifact using (https://github.com/flatironinstitute/NoRMCorre) and then processed them. Neuropil decontamination was performed subtracting from each ROI the time course of the average fluorescence intensity measured in visually identified neuropil region multiplied by a factor α = 0.7 (Chen et al., 2013). For SLS series, we extracted the fluorescence activity of each pixel in the SLS and then used a strategy based on dimensionality reduction to detect large motion artifacts. We reduced the dimensionality of our data by projecting each data point onto the first principal component (PC1, the one with the largest variance). PC1 accounted, on average across sessions, for 15.5 ± 9.2% of the variance of the data. The algorithm we used to compute PCA was the standard one based on single value decomposition and implemented in the MATLAB function “pca.” In more detail, we considered the time vector of the first term in the SVD of the [num SLS pixel] x [num frames] raster and assumed that neural activity was characterized only by smooth variations in the SLS raster. We fitted the time vector using an autoregressive model with order p = 2 (AR(2)) (Pnevmatikakis et al., 2016) and computed the correlation between the time vector and its fit using a sliding window of ∼10 s (the sliding window was moved in the temporal axis by one frame at a time). If the correlation coefficient dropped below a threshold value set to 0.3, a large motion artifact was detected and the line scan acquisition was discarded from the time of detection of the artifact to the end of the SLS acquisition. For SLS acquisitions, the neuropil signal was subtracted using three different strategies. In the first, we implemented a neuropil subtraction strategy based on pixels labeled as background. We considered as neuropil activity the time vector of the first term in the SVD of the [num global np px] x [num frames] raster, we multiplied this time vector by a constant factor (0.7; Chen et al., 2013), and we subtracted the resulting value from the activity of each ROI. This first component accounted for the largest variability in background pixels (fraction of variance explained by first component 12.4 ± 7.7%). In the second method, we implemented a local neuropil decontamination strategy similarly to Chen et al. (2013). For each ROI, we considered as neuropil activity the time vector of the first term in the SVD of the [num local np px] x [num frames] raster (fraction of variance explained by first component 33.4 ± 15.1%), we multiplied this time vector by a constant factor (0.7; Chen et al., 2013), and we subtracted the resulting value from the activity of the ROI. Given the limited number of pixels in the surround, this second method could be implemented only with trajectories with surround > 1 pixel. In the third method, we applied a neuropil decontamination strategy based on PCA of the ROIs signal, instead of the single pixels signal. We computed the [num ROIs] x [num frames] raster, by averaging the activity of pixels assigned to the same ROI (and to its surround). Therefore, each row in the raster represents the fluorescence activity of a ROI. We considered as neuropil activity the time vector of the first term in the SVD of this raster (fraction of variance explained by first component 55.9 ± 12.1%). The coefficient relative to each ROI in the first principal score represents the ROI’s projection on the first principal direction used for the decomposition. In this third case, for each ROI we used this coefficient as a weight for the neuropil activity and then subtracted the weighted neuropil from the ROI’s activity. All considered neuropil decontamination strategies resulted in decreased pairwise correlation in the estimated fluorescence trace of ROIs acquired with SLS.

To compensate for potential small movement artifacts within each ROI + surround region, we reassigned pixels based on their SNR value after the neuropil decontamination. If N pixels were originally identified during the SLS trajectory identification, we searched for the N pixels with highest SNR within the ROI + surround region in a sliding window of 10 s (the sliding window was moved in the temporal axis by one frame at a time) and we considered only these reassigned pixels for further processing.

##### Segmentation and ROIs detection

For raster scan series, the segmentation of the FOV was done with the semi-automatic procedure described above. For SLS series, to assign pixels to relevant cellular areas, we used the ROIs obtained in the segmentation process used to identify the SLS trajectory (see above). Pixels were assigned to one of the following three groups: *i)* pixels belonging to ROIs (if the distance between the pixel and a reference ROI was smaller than 1 pixel); *ii)* pixels in the surround of a ROI (if the distance between the pixel and a reference ROI was larger than 2 pixels and smaller than 4 pixels); *iii)* pixels in the background (if the distance between the pixel and all reference ROIs was larger than 4 pixels). If a pixel fell into more than one group or did not belong to any class, that pixel was not assigned to any of the groups.

##### Calcium activity extraction and events detection

Fluorescence traces were displayed as non-negative raw signal, ΔF(t)/F_0_ = (F(t)-F_0_)/F_0_ (where F(t) is the fluorescence at time t, F_0_ is the fluorescence intensity in periods of no activity and negative values of ΔF(t)/F_0_ are set to 0) or processed using NMF and deconvolution (https://github.com/flatironinstitute/CaImAn-MATLAB). For each ROI fluorescence values below the 25^th^ percentile of the average trace are regarded as periods of no activity (Sun et al., 2016). Values from all pixels in a given ROI were averaged to obtain fluorescence signal of that ROI. Detection of fluorescence events was performed on ΔF/F_0_ traces. A fluorescence event was detected at time t if: *i)* it occurred outside the baseline periods; *ii)* the fluorescence amplitude at time t was above the 50^th^ percentile of average ROI signal; *iii)* the time to peak was compatible with GCaMP6s dynamics. The time of the fluorescence event was set as the time of the fluorescence peak. Stimulus-evoked fluorescence events (Figures 5, 6, and 7) were detected in a response window of 2 s following the stimulus and the baseline fluorescence in a 7 s continuous no activity period preceding the sensory stimulus. Fluorescence events were classified as large or small based on whether their amplitude was higher or respectively lower than a threshold value placed at the 50^th^ percentile of their amplitude distribution.

In combined imaging and electrophysiological recordings (Figure 5), isolated APs (and associated fluorescence events) were defined as those APs which were preceded or followed by a period of no activity (no APs) of 0.4 s and 1 s, respectively. In electrophysiological recordings, traces were high-pass filtered (corner frequency, 100 Hz) and APs were detected based on a threshold criterion set at two times the standard deviation of trace average value in a 10 s period of no activity. Precise temporal alignment of electrophysiological recordings, imaging data, and sensory stimulation was achieved through TTL signals.

The SNR was defined as the absolute maximum fluorescence value in each ROI from a given t-series (from either raster scan or SLS) divided by the standard deviation of the fluorescence values of all the no activity periods. In the case of air puff whisker stimulation, a cell was considered responsive if at least one fluorescence event was detected in the stimulus presentation window for at least one pulse of the train (see above for whisker stimulation protocol). The accuracy in the detection of isolated APs was calculated as the ratio between the number of detected isolated APs in the juxtasomal electrophysiological recordings and the number of detected corresponding fluorescence events.

##### Graphical user interface (GUI)

The t-series processing was done using a custom-made graphical user interface (GUI). The GUI was written in MATLAB (R2017b, The MathWorks, Inc., Natick, Massachusetts, United States). The GUI allowed to: *i)* import .tiff sequence (raw data) acquired both with raster scan and SLS, browse frame by frame the series or display temporal projections (average, absolute maximum, temporal pixel autocorrelation or local dynamic range in adjustable frame intervals); *ii)* correct for motion artifacts in raster scan acquisition using the algorithms provided in https://github.com/flatironinstitute/NoRMCorre; *iii)* perform the semi-automatic segmentation procedure described above; *iv)* perform NMF-based segmentation using the algorithms provided in https://github.com/flatironinstitute/CaImAn-MATLAB; *v)* compute the SLS trajectories controlling for the inclusion of surrounding regions; *vi)* save relevant files.

#### Colocalization analysis

The manual selection of ROIs in each FOV was performed in Fiji (ImageJ V. 1.52e; Schindelin et al., 2012). This was done for ROI identification including whole cellular profile, nuclear, and cytoplasmic regions. To perform colocalization analysis, we segmented each FOV using the semi-automatic SNR pixel-wise approach. We divided each of 8 long t-series from 8 FOV in 4 short t-series, resulting in 32 short t-series. The segmentation process returned a binary image for each t-series. Pixels belonging to ROIs in each short t-series were assigned the value ‘1’, while pixels belonging to the remaining FOV were given the value ‘0’. Colocalization was calculated summing the values of individual pixels across consecutive short t-series. If a pixel belonged to the selected ROI in all 4 epochs, the sum of its values across the short t-series was 4 and at that pixel was assigned 100% of colocalization. Accordingly, when a pixel belonged to the ROI in 1, 2 or 3 t-series, colocalization was set at 0%, 50%, 75%, respectively.

#### Ensembles detection

We computed neuronal ensembles similarly to Miller et al. (2014) and the code provided with the FluoroSNNAP software (Patel et al., 2015) (https://www.seas.upenn.edu/∼molneuro/software.html). As described in Miller et al. (2014), ensembles are defined as groups of neurons simultaneously coactive in a given frame. To detect frames in which a given ROI was active we used raw fluorescence traces processed as described in Pnevmatikakis et al. (2016) to obtain signal denoising and NMF processed traces. Periods (frames) of activity for each ROI was defined using a threshold criterion on the amplitude of NMF-processed trace setting as “active” every frame in which NMF trace was higher than 3 s.d. above 0. Traces were then binarized assigning value 0 to all periods (frames) where the processed traces were below threshold and value 1 otherwise. The result is an “activity trace,” a series of frames assigned as “active” or “inactive.” Whenever at least two ROIs were “active” in the same frame, we tested for the detection of an ensemble event. To do this we generated 1,000 surrogate distribution of the “activity trace” for each ROI randomly shuffling frames assigned as “active” and “inactive.” A threshold for ensemble detection was set as the number of coactive cells exceeding only 5% of the surrogate “activity traces.” In order to compute neural ensembles on down sampled datasets, we first detected events on the original traces and then binned the activity (bin width = 0.5 s) for both raster and SLS acquisitions assigning a value of 1 to bins where at least one event was present, 0 otherwise. We repeated the neuronal ensembles extraction on the binned datasets.

#### NMF and Variance Accounted For

We applied spatial NMF (Lee and Seung, 1999) to either the entire normalized calcium activity or to the normalized calcium activity restricted to those time instants where an ensemble was detected. We decomposed the spatial patterns of activity across the ROIs at any instant of time, or at any instant of time where an ensemble was detected, (which is a non-negative dataset) into a given number of non-negative modules with non-negative coefficients, as follows:

F=M∗A+E

where F is the normalized calcium activity in each of the ROIs at time instant t, M are the spatial modules (each being a pattern of normalized calcium activity in each ROI), A is the set of spatial activation coefficients in each ROI in the considered instant of time, and E the residual error.

The number of modules is a free parameter of the factorization, which expresses the number of possible different firing patterns in which we factorize the data. We repeated the factorization by varying the number of modules from the minimal possible value (1) to the maximal possible value (the number of ROIs in the FOV). For each value of the possible number of modules in this range, and for each FOV, we computed the percentage of Variance Accounted For (VAF) of the NMF decomposition, defined as:

$$VAF=100*\left(1-\frac{\underset{i}{\sum }\sum_{j}e_{ij}^{2}}{\underset{i}{\sum }\sum_{j}f_{ij}^{2}}\right)$$

To characterize the spatial sparseness of each spatial module we used a sparseness index defined as:

$$s=\frac{\sqrt{\sum_{j}w_{j}^{2}}}{\sum_{j}w_{j}}$$

Where $w_{j}$ represents the weight of the j-th ROI to the module. The sparseness index reaches its maximal value of 1 if only 1 ROI participate to a module.

#### Support vector machine

We trained a support vector machine (SVM) classifier (Bzdok et al., 2018) using non-balanced dataset to test if calcium population dynamics could be used to infer the presence/absence of whisker stimulation in SLS and raster scan t-series. Chance level was set to be proportional to the number of “baseline” frames (without whisker stimulation) over the total number of frames. The dataset was split in 50% training set and 50% test set. Only series with at least half of the ROIs showing calcium events in at least half of the whisker stimuli were considered. On the training set 10-fold cross validation was performed to select the optimal parameters for the decoder. We used the n-dimensional array of the neural population activity (n = number of ROIs) to decode the presence of the air puff. The accuracy of the SVM classifier was given by the percentage of correctly predicted states (presence/absence of whisker stimulation).

### Quantification and Statistical Analysis

Values are expressed as mean ± sd; the number of samples (N) and p values are reported in the figure legends or in the text. No statistical methods were used to pre-determine sample size, but sample size was chosen based on previous studies (Chen et al., 2013, Miller et al., 2014). All recordings with no technical issues were included in the analysis and blinding was not used in this study. Quantification was performed as described in previous paragraphs. Statistical analysis was performed with MATLAB software (Mathworks, Natick, MA) and Origin(Pro) 2018 (Origin OriginLab, Northampton, MA). A Kolmogorov-Smirnov test was run on each experimental sample to test for normality. The significance threshold was always set at 0.05. When comparing two paired populations of data, paired t test or paired Wilcoxon signed-rank test were used to calculate statistical significance in case of normal and non-normal distribution, respectively. Unpaired t test and two-sample Kolmogorov-Smirnov test or Wilcoxon rank-sum test were used for unpaired comparisons of normally and non-normally distributed data, respectively. Fisher’s exact test was used to compare set of pixels selected in long versus short t-series. All tests were two-sided, unless otherwise stated. Analysis of variance, functional modules identification, modules sparseness and SVM classification were performed on MATLAB software (Mathworks, Natick, MA) using custom written codes.

### Data and Code Availability

The dataset/code supporting the current study are available from the corresponding author on request.

## Supplemental Information

Document S1. Figures S1–S9

Document S2. Article plus Supplemental Information
