# Supplementary material for: High-Accuracy Detection of Neuronal Ensemble Activity in Two-Photon Functional Microscopy Using Smart Line Scanning
Source: Cell Rep. 2020 Feb 25;30(8):2567–2580.e6. doi: 10.1016/j.celrep.2020.01.105 (PMC7043026; doi:10.1016/j.celrep.2020.01.105)
Supplement: Document S1. Figures S1–S9 [file mmc1.pdf]

Cell Reports, Volume *30*

Supplemental Information

High-Accuracy Detection of Neuronal Ensemble Activity in Two-Photon Functional Microscopy Using Smart Line Scanning

Marco Brondi, Monica Moroni, Dania Vecchia, Manuel Molano-Mazón, Stefano Panzeri, and Tommaso Fellin

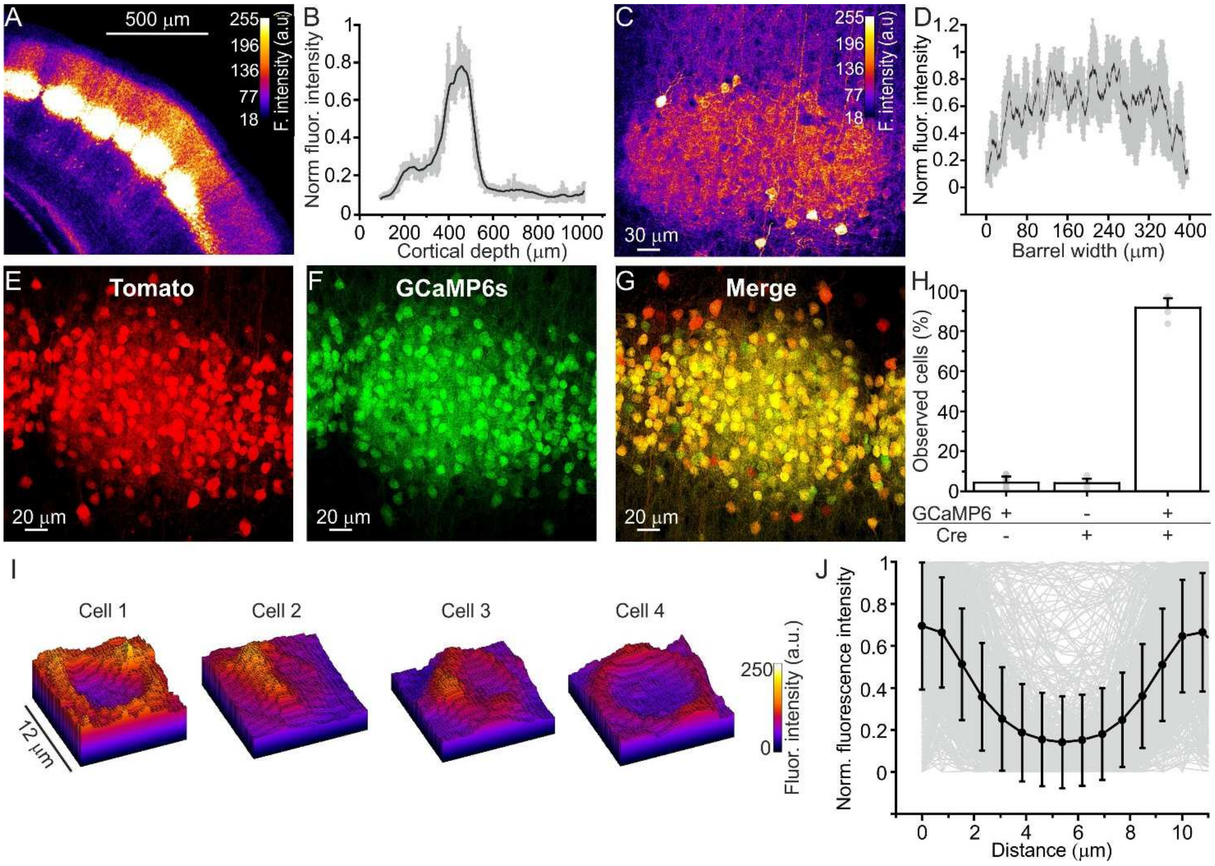

Figure S1. Selective expression of GCaMP6s in layer IV principal neurons of the mouse barrel cortex. Related to Figure 2. **(A)** Two-photon image showing a coronal section of the barrel cortex of a Scnn1a-cre mouse injected with AAV1.Syn.Flex.GCaMP6s. **(B)** Normalized fluorescence intensity as a function of cortical depth. The grey lines represent standard deviation, the dark line represents the average across FOVs (N = 4). **(C)** Two-photon image showing a zoom on a single barrel. **(D)** Normalized fluorescence intensity as a function of the barrel width (N = 4). **(E-G)** Two-photon image showing TdTomato expression (left panel, red), GCaMP6 (middle panel, green), and the merge in a coronal section of the barrel cortex from a bigenic Scnn1a-cre mouse x Floxed-TdTomato mouse injected with AAV1.Syn.Flex.GCaMP6s. **(H)** Percentage of observed cells under the different experimental conditons (N = 4 mice). **(I)** 3D intensity profile for four representative layer IV cells expressing GCaMP6s *in vivo*. **(J)** Fluorescence intensity profile along the cell's diameter (N = 604 neurons from 8 mice). Grey lines indicate individual cells; the black line indicates the average across neurons ± sd.

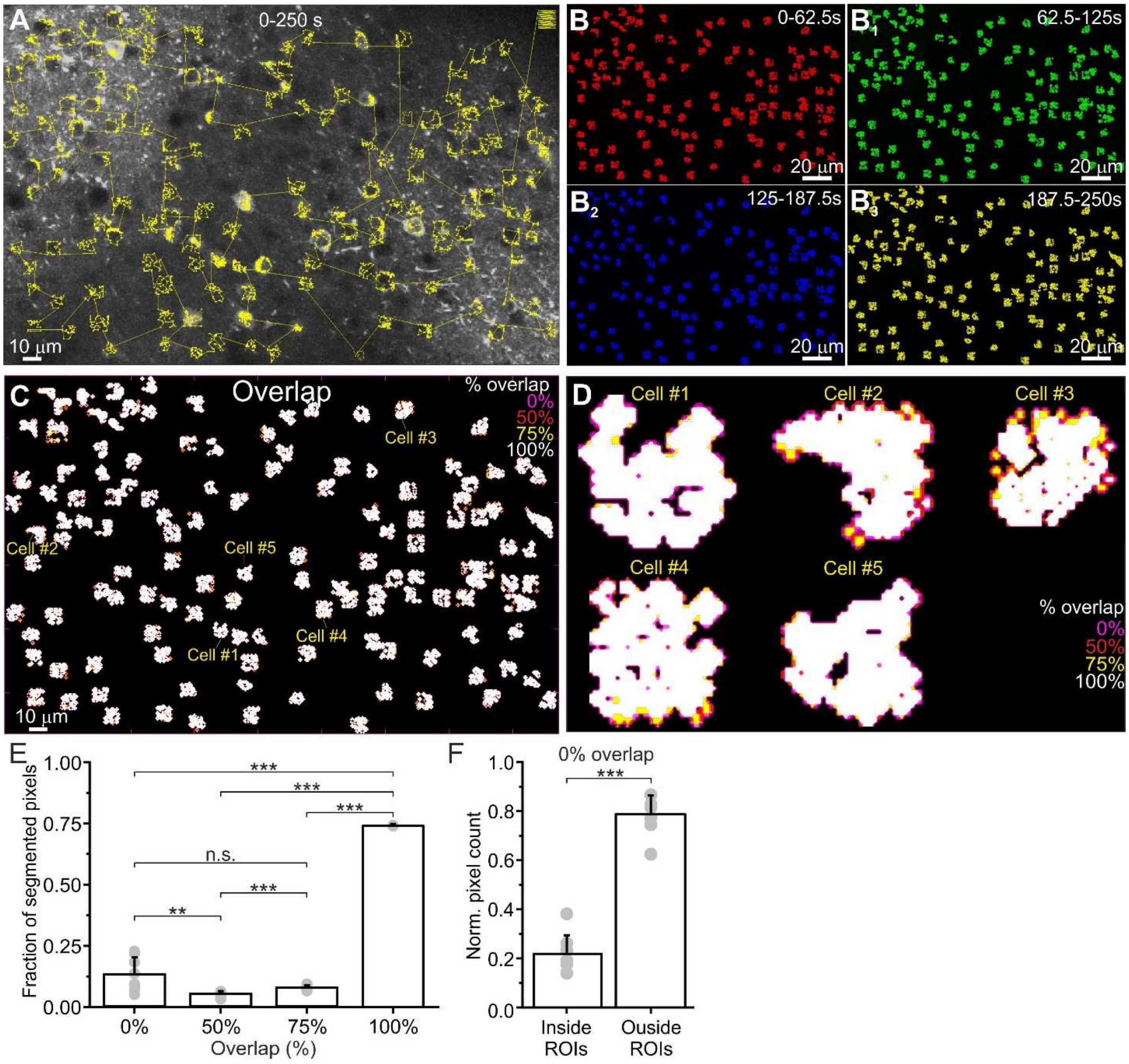

Figure S2. Computation of SLS trajectories is stable over time. Related to Figure 2. **(A)** Two-photon raster scanning image (single frame from a 250 seconds long time series) of layer IV neurons *in vivo*. The SLS trajectory is depicted in yellow. **(B-B$_3$)** SLS trajectories designed on short t-series obtained dividing the t-series shown in (A) into four sequential epochs: 0-62.5 s in (B), 62.5-125 s in (B$_1$), 125-187.5 s in (B$_2$) and 187.5-250 s in (B$_3$). **(C)** Overlap between the SLS trajectories shown in (B-B$_3$). Only pixels belonging to cells of interest are displayed. Colors represent the percentage of overlap between the selected pixels in the four epochs: 0 % (magenta), 50 % (red), 75 % (yellow) and 100 % (white), respectively. **(D)** Zoom in of the five cells highlighted in (C). **(E)** Normalized distribution of segmented pixels showing 0 %, 50 %, 75 % or 100 % colocalization. N = 8 groups of 4 consecutives short t-series from 8 FOV in 8 animals; two-sample KS test, p = 2E-3 between 0 % and 50 % colocalization; p = 2E-4 between 50 % and 75 % colocalization; p = 2E-4 between 75 % and 100 % colocalization; p = 2E-4 between 0 % and 100 % colocalization; p = 0.08 between 0 % and 75 % colocalization; p = 2E-4 betwenn 50 % and 100 % colocalization. In this as well in other figures: *, $p < 0.05$; **, $p < 0.01$; ***, $p < 0.001$; n.s., non significant. Grey dots indicate individual measurements, the black bars indicate the average across measurements ± s.d. **(F)** Normalized number of segmented pixels with no colocalization (0 % in F) that belonged to ROIs (Inside ROIs) or that belonged to the segments connecting adjacent ROIs (Outside ROIs). N = 8 groups of 4 consecutives short t-series from 8 FOVs in 8 animals for both Inside ROIs and Outside ROIs conditions, respectively, two-sample KS test, p = 2E-4.

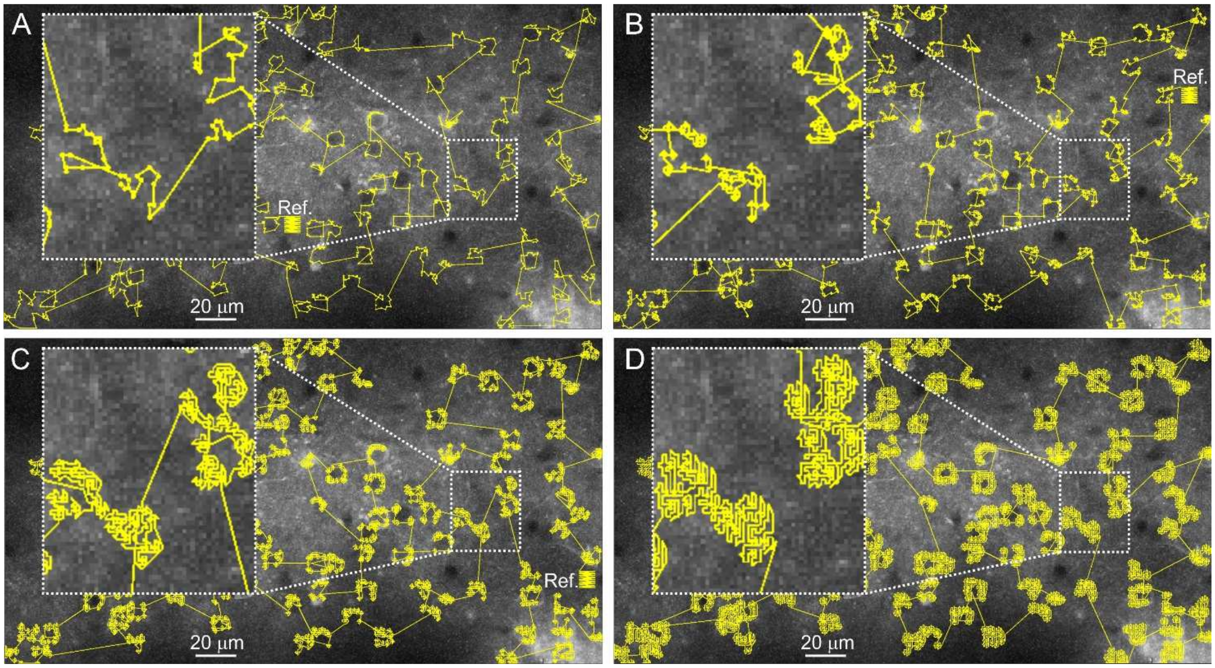

Figure S3. Generation of SLS trajectories. Related to Figure 2. **(A)** Two-photon image of layer IV GCaMP6-expressing neurons. The yellow line represents the trajectory generated by SLS algorithm. Note that a reference box (Ref.) can be included in the SLS to monitor x, y motion artefacts. The region of the FOV highlighted in white is shown at an enlarged scale in the inset. **(B-D)** Same as in (A) for SLS trajectories with various surround regions (one pixel in B, two pixels in C, and three pixels in D).

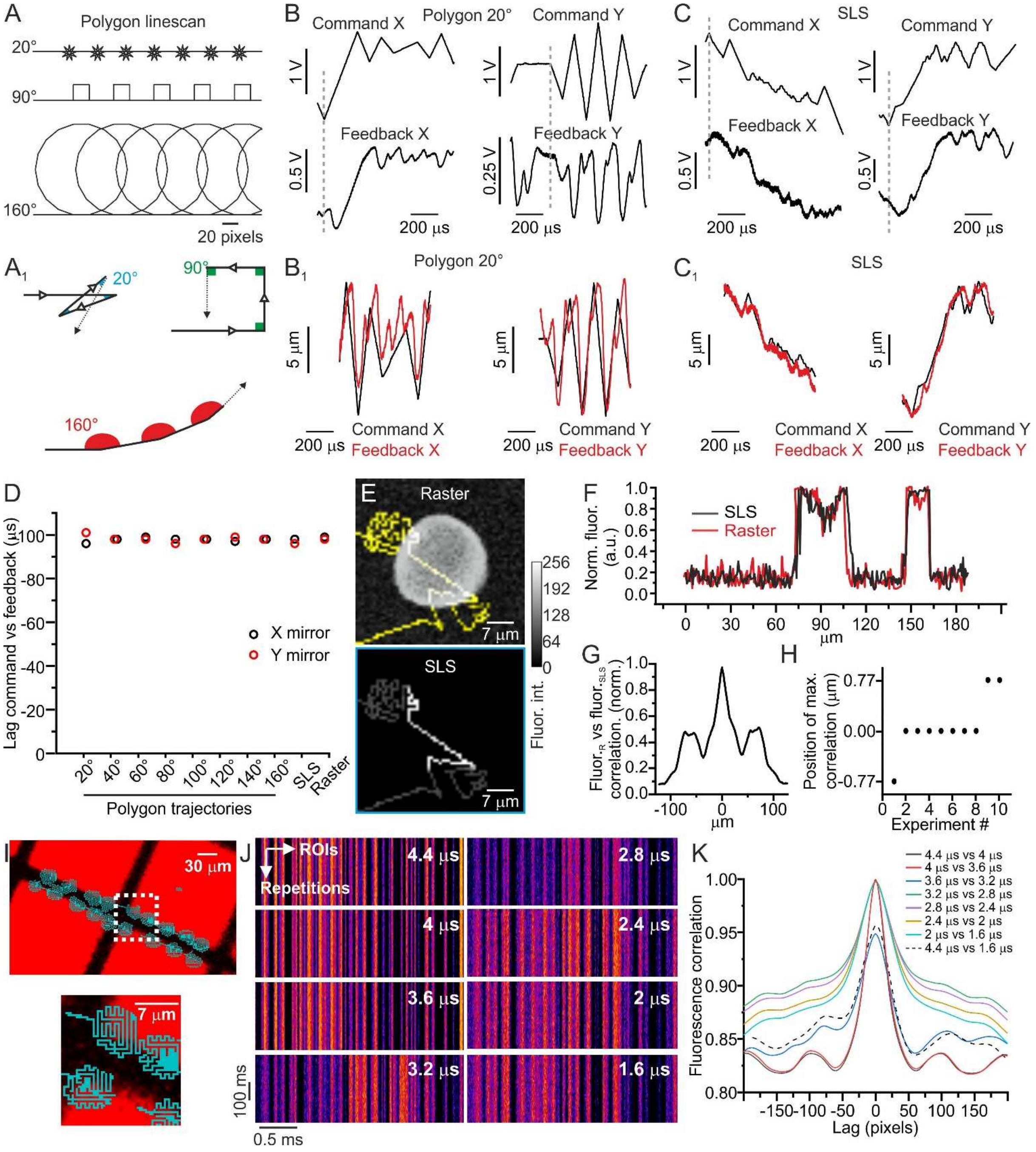

Figure S4. Precision of mirror positioning. Related to Figure 2. **(A)** Polygon line trajectories used to measure the temporal lag between the command signal and feedback signals of galvanometric mirrors as a function of the angle between successive segments of the line trajectory. **(A$_1$)** Zoom in of the polygon trajectories shown in (A). **(B)** Left: Command (top) and feedback (bottom) signal for the X galvanometric mirror during a polygon trajectory with 20° angle. Right: Command (top) and feedback (bottom) signal for the Y galvanometric mirror during the same polygon trajectory shown on the left. **(B$_1$)** Command (black) and feedback (red) signals are shown overlapped for the X (left) and Y (right) direction during a 20° polygon line scanning. Please note that the feedback signal was shifted backward in time by the time lag between command and feedback signals. **(C-C$_1$)** Same as in (B-B$_1$) for a typical SLS trajectory. **(D)** Temporal lag between the command signal and the feedback signal of the galvanometric mirrors during polygon line trajectories, SLS trajectories (SLS), and raster scanning (Raster). **(E)** Top: Two-photon fluorescence image showing a pollen grain obtained in raster scanning (greyscale signal) and an overlaying SLS trajectory (yellow line). Bottom: same field of view as above showing only the intensity of the pixels of the SLS (greyscale signal). **(F)** Normalized fluorescence intensity of pixels belonging to the SLS trajectory shown in the bottom panel of (E) and the pixels in the same spatial position but

recorded in the raster scanning image shown in the upper panel of (E). **(G)** Cross-correlogram between the two signals shown in (F). **(H)** Time of peak cross correlation across different experiments. **(I)** A SLS trajectory (cyan) is projected onto a fluorescent grid (red). The fine spatial structure of the SLS trajectory is shown at an expanded scale in the inset. **(J)** SLS acquisitions with the trajectory shown in (I) as a function of the acquisition dwell time (from 4.4 μs to 1.6 μs). **(K)** Correlation between fluorescent signals recorded with SLS at different acquisition dwell times.

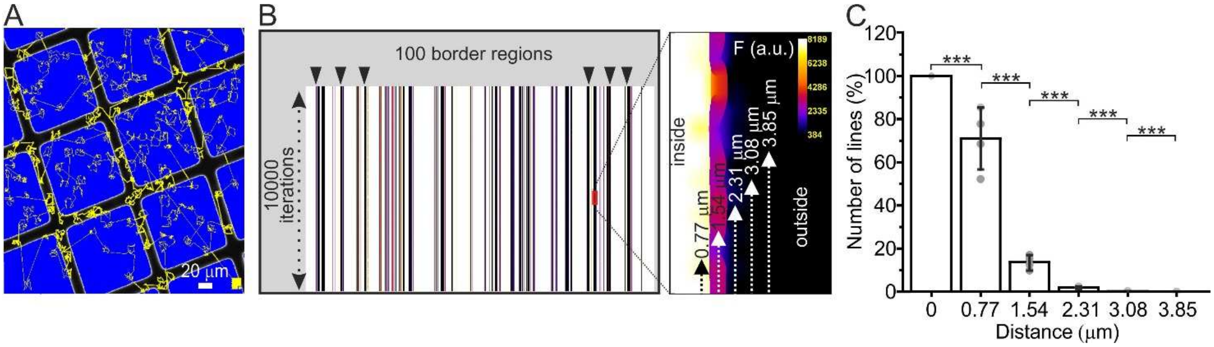

Figure S5. Stability of line scan trajectories across scanning trials. Related to Figure 2. **(A)** A line scan trajectory used for *in vivo* recording (yellow line) is scanned on a fluorescent grid (blue squares, 120 µm period). ROIs are randomly positioned inside fluorescent region (blue squares) or in non fluorescent regions (dark areas) or at the border between the fluorescent and the non fluorescent region. Pixel size of SLS acquisitions is 0.77 µm. **(B)** A SLS is iterated 10,000 times on the grid in four different days (total scanned trajectories: 40,000). Fluorescence intensity of each pixel is displayed on a logarithmic pseudocolor scale. Based on the position of the scanned pixel with respect to the grid, we defined 100 border regions. Inset: magnification of a set of the 15 SLS repetitions around one border region (highlighted in red in the left panel). No bleaching of the grid fluorescence was observed across 10,000 repetitions. **(C)** Number of SLS acquisitions with fluorescence intensity higher than noise across identified border regions as a function of the distance from the highly fluorescent region (see STAR Methods for details). Pixel size of SLS acquisitions is 0.77 µm. The average across 4 sequences of 10,000 SLS trajetories is displayed in black, values of individual sequences is shown in grey. Paired *t*-test for 0 µm *vs* 0.77 µm p = 1E-7, for 0.77 µm *vs* 1.54 µm p = 2E-8, for 1.54 µm *vs* 2.31 µm p = 6E-9, for 2.31 µm *vs* 3.08 µm p = 7E-12, and for 3.08 µm *vs* 3.85 µm p = 2E-8.

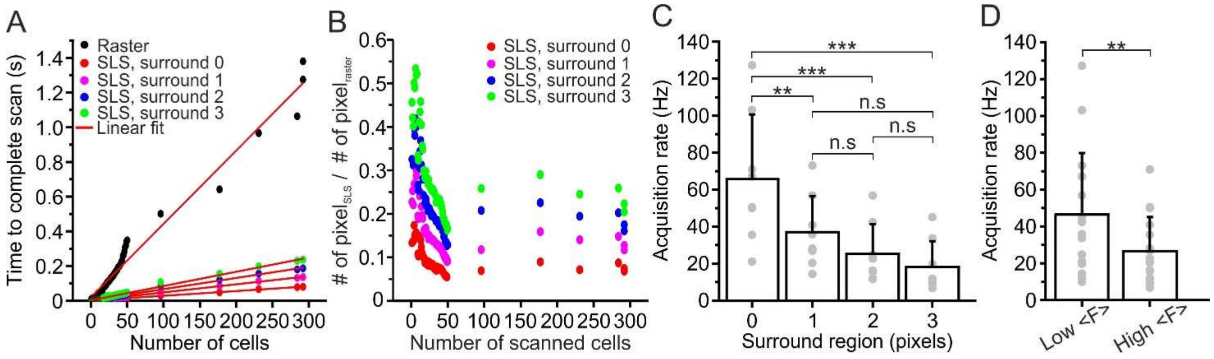

Figure S6. Acquisition speed in SLS. Related to Figure 2. **(A)** Time required to complete a scan as a function of the number of scanned cells for raster scan (black circles) and SLS (colored circles). In each SLS the number of pixels *per* cell was mantained constant while the surround region was increased from 0 to 3 pixels (red, pink, blue, and green dots). Red lines indicate the linear fit of data. **(B)** Ratio between the number of pixels in each SLS trajectory and the number of pixels in raster scan image (# pixel_{SLS} /# pixel_{raster}) as a function of the number of scanned cells. **(C)** Acquisition rate of SLS as a function of number of pixels included in the surround region (S0, S1, S2, and S3). N = 8 SLS acquisitions from 8 FOVs. Paired *t*-test, p = 0.009 between S0 and S1 , p = 6E-4 between S0 and S2, p = 6E-4 between S0 and S3, p = 0.21 between S1 and S2, p = 0.053 between S1 and S3, p = 0.36 between S2 and S3. **(D)** Acquisition frame rate of SLS in 4 FOVs characterized by low (Low <F>) and high (High <F>) GCaMP6 fluorescence *in vivo*. N = 16 SLS acquisitions from 4 FOVs for both Low <F> and High <F>. Paired *t*-test, p = 0.003.

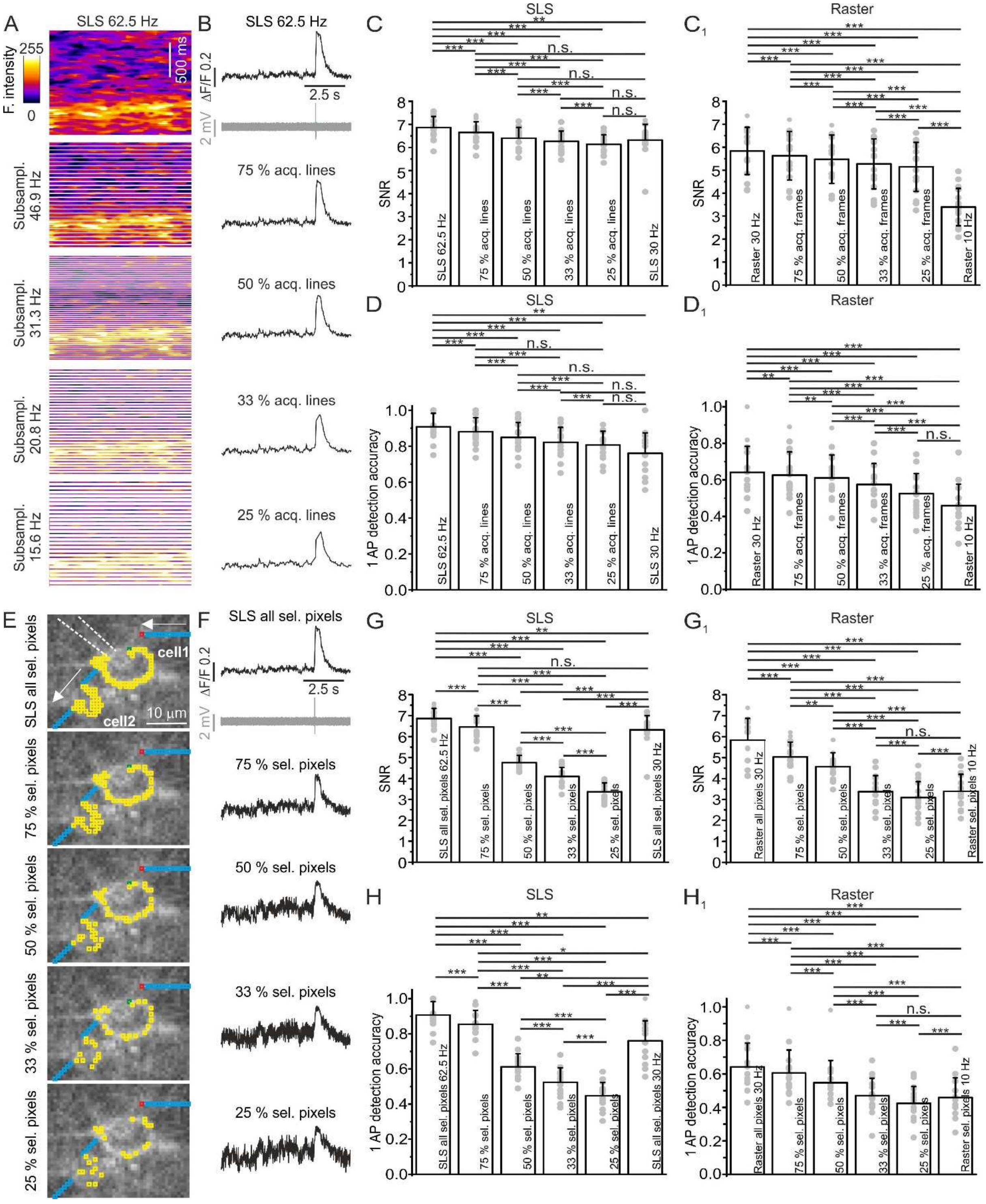

Figure S7. The SNR and the accuracy in detecting single APs depend on both the acquisition rate and the dwell time *per* cell. Related to Figure 5. **(A)** Top: representative SLS acquisitions at 62.5 Hz from one cell in combined imaging and juxtasomal electrophysiological recordings (see Figure 5). Bottom: SLS acquisitions were temporally down sampled maintaining 75 %, 50 %, 33 %, and 25 % of the acquired SLS. White horizontal stripes indicates the position of the removed SLS acquisitions. **(B)** ΔF/F (black) over time for the cell shown in (A), which was simultaneously recorded in the juxtasomal electrophysiological configuration (grey trace), for SLS at 62.5 Hz (top) and after temporal down sampling (bottom traces). **(C)** SNR of fluorescence signals in SLS acquisitions in which one single spike was observed in simultaneous juxtasomal electrophysiological recordings as a function of the temporal down sampling. Grey dots, N = 16 cells from 8 FOV in 4 animals. Error bars represent standard deviation. Paired sample Wilcoxon signed rank test: SLS

62.5 Hz vs 75% p = 7.1E-4, SLS 62.5 Hz vs 50 % p = 4.8E-4, SLS 62.5 Hz vs 33 % p = 4.8E-4, SLS 62.5 Hz vs 25 % p = 5.8E-4, SLS 62.5 Hz vs SLS 30 Hz p = 0.002; 75 % vs 50 % p = 7.3E-4, 75 % vs 33 % p = 4.8E-4, 75 % vs 25 % p = 0.072, 75 % vs SLS 30 Hz p = 0.003; 50 % vs 33 % p = 7.2E-4, 50 % vs 25 % p = 4.8E-4, 50 % vs SLS 30 Hz p= 0.064; 33 % vs 25 % p = 3.1E-4, 33 % vs SLS 30 Hz p = 0.083. 25 % vs SLS 30 Hz p = 0.17. **(C_1)** Same as in (C) but for raster scanning acquisitions. Raster scanning acquisitions were temporally down sampled maintaining 75 %, 50 %, 33 %, and 25 % of the acquired frames. N = 16 cells from 8 FOV in 4 animals. Paired sample Wilcoxon signed rank test: Raster 30 Hz vs 75 % p =4.8E-4, Raster 30 Hz vs 50 % p =4.8E-4, Raster 30 Hz vs 33 % p = 4.8E-4, Raster 30 Hz vs 25 % p = 4.8E-4, Raster 30 Hz vs Raster 10 Hz p = 4.8E-4; 75 % vs 50 % p = 0.007, 75 % vs 33 % p = 4.8E-4, 75 % vs 25 % p = 4.8E-4, 75 % vs Raster 10 Hz p = 4.8E-4; 50 % vs 33 % p = 4.8E-4, 50 % vs 25 % p = 4.8E-4, 50 % vs Raster 10 Hz p = 4.8E-4; 33 % vs 25 % p = 4.8E-4, 33 % vs Raster 10 Hz p = 4.8E-4; 25 % vs Raster 10 Hz p = 0.48. **(D)** Accuracy in detecting single AP for the SLS experiments shown in (C). N = 16 cells from 8 FOV in 4 animals. Paired sample Wilcoxon signed rank test: SLS 62.5 Hz vs 75 % p = 7.1E-4, SLS 62.5 Hz vs 50 % p = 4.8E-4, SLS 62.5 Hz vs 33% p = 4.8E-4, SLS 62.5 Hz vs 25 % p = 5.8E-4, SLS 62.5 Hz vs SLS 30 Hz p = 0.002; 75 % vs 50 % p = 7.3E-4, 75 % vs 33 % p = 4.8E-4, 75 % vs 25 % p = 0.072, 75 % vs SLS 30 Hz p = 0.003; 50 % vs 33 % p = 7.2E-4, 50 % vs 25 % p = 4.8E-4, 50 % vs SLS 30 Hz p = 0.064; 33 % vs 25 % p = 3.1E-4, 33 % vs SLS 30 Hz p = 0.083; 25 % vs SLS 30 Hz p = 0.17. **(D_1)** Same as in (D) but for raster scanning acquisitions. N = 16 cells from 8 FOV in 4 animals. Paired sample Wilcoxon signed rank test: Raster 30 Hz vs 75 % p =4.8E-4, raster 30 Hz vs 50 % p = 4.8E-4, raster 30 Hz vs 33 % p = 4.8E-4, raster 30 Hz vs 25% p = 4.8E-4, raster 30 Hz vs raster 10 Hz p = 4.8E-4; 75 % vs 50 % p = 0.006, 75 % vs 33% p = 4.8E-4, 75 % vs 25 % p = 4.8E-4, 75 % vs raster 10 Hz p = 4.8E-4; 50 % vs 33 % p = 4.8E-4, 50 % vs 25 % p = 4.8E-4, 50 % vs raster 10 Hz p = 4.8E-4; 33 % vs 25 % p = 4.8E-4, 33 % vs raster 10 Hz p = 4.8E-4; 25 % vs raster 10 Hz p = 0.48. **(E)** Top: Two-photon image showing a GCaMP6s expressing neuron *in vivo* which was imaged with a SLS trajectory (cyan line) and simultaneously recorded with a glass pipette (dashed white lines). Yellow dots indicate the pixels of the SLS trajectory belonging to the cell. Bottom panels: pixels belonging to cells were randomly down sampled maintaining 75 %, 50 %, 33 %, and 25 % of the acquired pixels (spatial down sampling). **(F)** ΔF/F (black) over time for one representative cell, which was simultaneously recorded in the juxtasomal electrophysiological configuration (grey trace), for SLS (top) and after spatial down sampling (bottom traces). **(G)** SNR of fluorescence signals in SLS acquisitions in which one single spike was observed in simultaneous juxtasomal electrophysiological recordings as a function of the spatial down sampling. Raster scanning acquisitions were randomly down sampled maintaining 75 %, 50 %, 33 %, and 25 % of the pixels within each ROI. Grey dots, N = 16 cells from 8 FOV in 4 animals. Error bars represent standard deviation. Paired sample Wilcoxon signed rank test: SLS all 62.5 Hz vs 75 % p = 4.8E-4, SLS all 62.5 Hz vs 50 % p = 4.8E-4, SLS all 62.5 Hz vs 33 % p = 4.8E-4, SLS all 62.5 Hz vs 25 % p = 4.8E-4, SLS all 62.5 Hz vs SLS all 30 Hz p = 0.007; 75 % vs 50 % p = 4.8E-4, 75 % vs 33 % p = 4.8E-4, 75 % vs 25 % p = 4.8E-4, 75 % vs SLS all 30 Hz p = 0.39; 50 % vs 33 % p = 4.8E-4, 50 % vs 25 % p = 4.8E-4, 50 % vs SLS all 30 Hz p = 5.8E-4; 33 % vs 25 % p = 4.8E-4, 33 % vs SLS all 30 Hz p = 5.8E-4; 25 % vs SLS all 30 Hz p = 4.8E-4. **(G_1)** Same as in (G) but for raster scanning acquisitions. N = 16 cells from 8 FOV in 4 animals. Paired sample Wilcoxon signed rank test: raster all 30 Hz vs 75 % p =4.8E-4, raster all 30 Hz vs 50 % p = 4.8E-4, raster all 30 Hz vs 33 % p = 4.8E-4, raster all 30 Hz vs 25 % p = 4.8E-4, raster all 30 Hz vs raster all 10 Hz p = 4.8E-4; 75 % vs 50 % p = 0.006, 75 % vs 33 % p = 4.8E-4, 75 % vs 25 % p = 4.8E-4, 75 % vs raster all 10 Hz p = 4.8E-4; 50 % vs 33 % p = 4.8E-4, 50 % vs 25 % p = 4.8E-4, 50 % vs raster all 10 Hz p = 4.8E-4; 33 % vs 25 % p = 4.8E-4, 33 % vs raster 10 Hz p = 0.48; 25 % vs raster 10 all 10 Hz p = 4.8E-4. **(H)** Accuracy in detecting single AP for the SLS experiments shown in (G). N = 16 cells from 8 FOV in 4 animals. Paired sample Wilcoxon signed rank test: SLS all 62.5 Hz vs 75 % p = 4.8E-4, SLS all 62.5 Hz vs 50 % p = 4.8E-4, SLS all 62.5 Hz vs 33 % p = 4.8E-4, SLS all 62.5 Hz vs 25 % p = 4.8E-4, SLS all 62.5 Hz vs SLS all 30 Hz p = 0.002; 75 % vs 50 % p = 4.8E-4, 75 % vs 33 % p = 4.8E-4, 75 % vs 25 % p = 4.8E-4, 75 % vs SLS all 30 Hz p = 0.012; 50 % vs 33 % p = 4.8E-4, 50 % vs 25 % p = 4.8E-4, 50 % vs SLS all 30 Hz p = 0.002; 33 % vs 25 % p = 4.8E-4, 33 % vs SLS all 30 Hz p = 4.8E-4; 25 % vs SLS all 30 Hz p = 4.8E-4. **(H_1)** Same as in (H) but for raster scanning acquisitions. N = 16 cells from 8 FOV in 4 animals. Paired sample Wilcoxon signed rank test: raster all 30 Hz vs 75 % p = 4.8E-4, raster all 30 Hz vs 50 % p = 4.8E-4, raster all 30 Hz vs 33 % p = 4.8E-4, raster all 30 Hz vs 25 % p = 4.8E-4, raster all 30 Hz vs raster all 10 Hz p = 4.8E-4; 75 % vs 50 % p = 7.2E-4, 75 % vs 33 % p = 4.8E-4, 75 % vs 25 % p = 4.8E-4, 75 % vs raster all 10 Hz p = 4.8E-4; 50 % vs 33 % p = 4.8E-4, 50 % vs 25 % p = 4.8E-4, 50 % vs raster all 10 Hz p = 4.8E-4; 33 % vs 25 % p = 4.6E-4, 33 % vs raster all 10 Hz p = 0.21; 25 % vs raster all 10 Hz p = 4.8E-4.

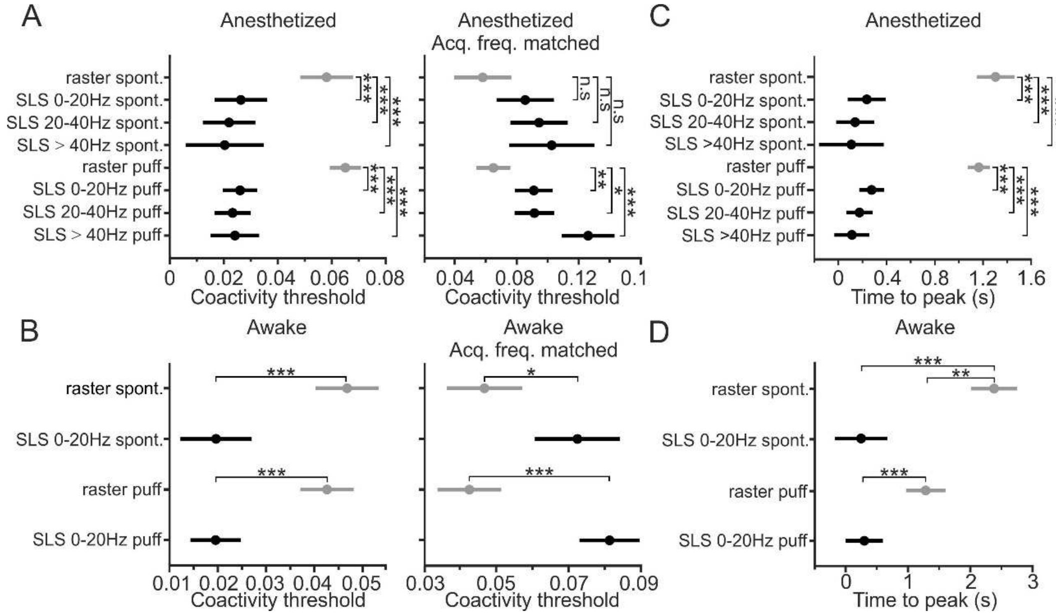

Figure S8. Decreased coactivity threshold of detected neural ensembles in SLS. Related to Figure 6. **(A)** Left: Coactivity threshold for ensemble activity detection in anesthetized animals under the different experimental conditions. Right: Coactivity threshold after down sampling of SLS series to match the acquisition rate of raster scanning series. Spontaneous activity: N = 6 for raster, N = 6 for SLS at 0-20 Hz, N = 6 for SLS at 20-40 Hz, N = 3 for SLS at > 40 Hz from 6 anesthetized mice. Air puff stimulation: N = 20 for raster, N = 15 for SLS at 0-20 Hz, N = 13 for SLS at 20-40 Hz, N = 7 for SLS at > 40 Hz from 6 anesthetized mice. Left, spontaneous activity: paired *t*-test raster *vs* SLS at 0-20 Hz p = 1E-4, raster *vs* SLS at 20-40 Hz p = 1E-5, raster *vs* SLS at > 40 Hz p = 1E-4. Air puff stimulation: paired *t*-test raster *vs* SLS at 0-20 Hz p = 6E-8, raster *vs* SLS at 20-40 Hz p = 6E-8, raster *vs* SLS at > 40 Hz p = 6E-8. Right, spontaneous activity: paired *t*-test raster *vs* SLS at 0-20 Hz p = 0.30, raster *vs* SLS at 20-40 Hz p = 0.056, raster *vs* SLS at > 40 Hz p = 0.056. Air puff stimulation: paired *t*-test raster *vs* SLS at 0-20 Hz p = 0.009, raster *vs* SLS at 20-40 Hz p = 0.012, raster *vs* SLS at > 40 Hz p = 1E-7. **(B)** Same as in (A) for experiments in awake mice. Spontaneous activity: N = 5 for raster and N = 4 for SLS at 0-20 Hz from 2 awake mice. Air puff stimulation: N = 7 for raster and N = 8 for SLS at 0-20 Hz from 2 awake mice. Left, spontaneous activity: paired *t*-test raster *vs* SLS at 0-20 Hz p = 1E-4. Air puff stimulation: paired *t*-test raster *vs* at SLS 0-20 Hz p = 4E-5. Right, spontaneous activity: paired *t*-test raster *vs* SLS at 0-20 Hz p = 0.019. Air puff stimulation: paired *t*-test raster *vs* SLS at 0-20 Hz p = 2E-5. **(C)** Time to peak measured for all isolated calcium events in anesthetized animals under the different experimental conditions. Spontaneous activity: N = 6 for raster, N = 6 for SLS at 0-20 Hz, N = 6 for SLS at 20-40 Hz, N = 3 for SLS at > 40 Hz from 6 anesthetized mice. Air puff stimulation: N = 20 for raster, N = 15 for SLS at 0-20 Hz, N = 13 for SLS at 20-40 Hz, N = 7 for SLS at > 40 Hz from 6 anesthetized mice. Spontaneous activity: paired *t*-test raster *vs* SLS at 0-20 Hz p = 6E-8, raster *vs* SLS at 20-40 Hz p = 6E-8, raster *vs* SLS at > 40 Hz p= 6E-8. Air puff stimulation: paired *t*-test raster *vs* SLS at 0-20 Hz p = 6E-8, raster *vs* SLS at 20-40 Hz p = 6E-8, raster *vs* SLS at > 40 Hz p = 6E-8. **(D)** Same as in (C) for awake mice. Spontaneous activity: N = 5 for raster, N = 4 for SLS at 0-20 Hz from 2 awake mice. Air puff stimulation: N = 7 for raster, N = 8 for SLS at 0-20 Hz from 2 awake mice. Spontaneous activity: paired *t*-test raster *vs* SLS at 0-20 Hz p = 2E-6. Air puff stimulation: paired *t*-test raster *vs* SLS at 0-20 Hz p = 8E-8. Paired *t*-test raster in spontaneous activity *vs* raster in air puff stimulation p = 0.001.

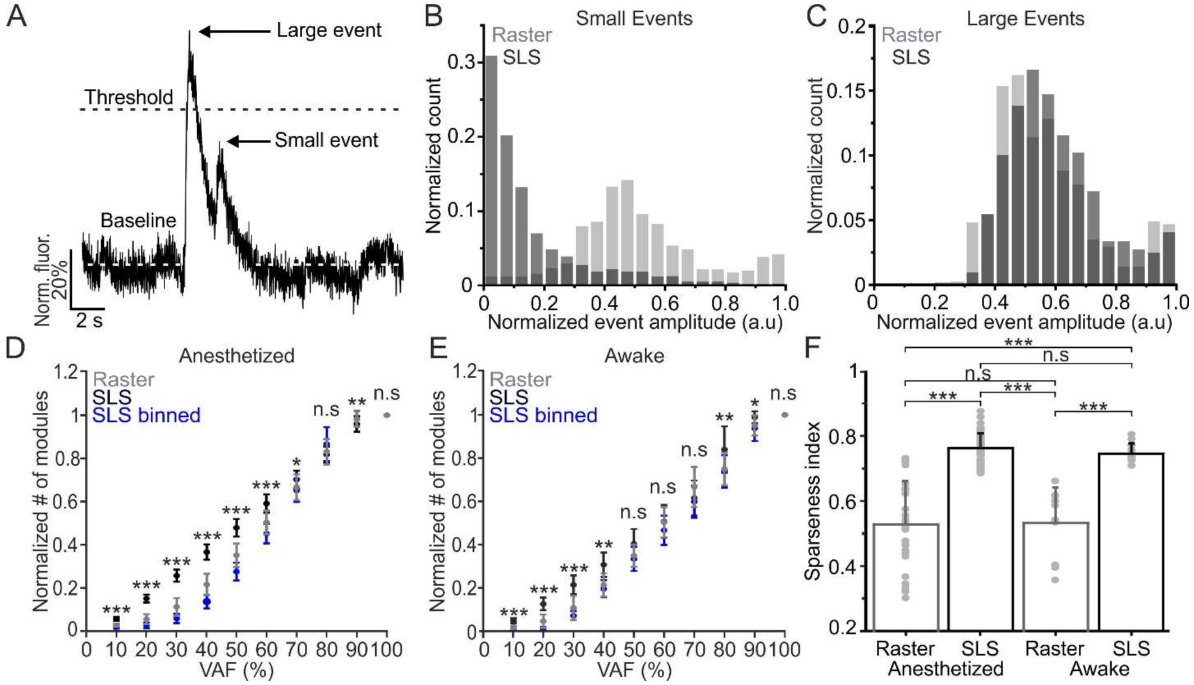

Figure S9. Increased number of small detected calcium events in SLS. Related to Figure 7. **(A)** Representative SLS fluorescence trace over time from one ROI showing two fluorescence events classified as small event (small) and large event (large) based on the value of a threshold set at the 50th percentile of the amplitude distribution of all detected calcium events. **(B)** Distribution of small calcium events detected from SLS (black) and raster (grey) acquisitions during spontaneous activity and air puff stimulation in anesthetized and awake animals. **(C)** Same as in (B) for large calcium events. **(D)** Normalized number of detected functional modules from ensemble activity as a function of the percentage of VAF. Data are reported for SLS acquisitions (black), raster acquisitions (grey), and SLS series down sampled to match the acquisition rate of raster scanning series (blue, SLS binned) in anesthetized mice. N = 26 for raster, N = 10 for SLS at 0-20 Hz, N = 6 for SLS at 20-40 Hz, N = 3 for SLS at > 40 Hz from 6 anesthetized mice. Comparison bewteen raster and SLS acquisitions: paired *t*-test, p = 3E-12 at 10% of VAF, p = 1E-12 at 20% of VAF, p = 1E-12 at 30% of VAF, p = 2E-12 at 40% of VAF, p = 2E-11 at 50% of VAF, p = 4E-8 at 60% of VAF, p = 0.025 at 70% of VAF, p = 0.072 at 80% of VAF, p = 0.003 at 90% of VAF, p = 1 at 100% of VAF. No significant p-values were obtained when comparing between raster and SLS binned at any percentage of VAF. **(E)** Same as in (D) for experiments in awake animals. N = 12 for raster, N = 12 for SLS at 0-20 Hz from 2 awake mice. Comparison bewteen raster and SLS acquisitions: paired *t*-test, p = 7E-5 at 10% of VAF, p = 1E-4 at 20% of VAF, p = 2E-4 at 30% of VAF, p = 0.001 at 40% of VAF, p = 0.078 at 50% of VAF, p = 0.88 at 60% of VAF, p = 0.12 at 70% of VAF, p = 0.008 at 80% of VAF, p = 0.01 at 90% of VAF, p = 1 at 100% of VAF. **(F)** Sparseness index of functional modules from ensemble activity in raster scan (N = 38) and SLS (N = 31) acquisitions from 6 anesthetized and 2 awake mice. Two sample KS test, p = 6E-14 between anesthetized raster and anesthetized SLS, p = 2E-7 between awake raster and awake SLS, p = 0.49 between anesthetized raster and awake raster, p = 1E-7 bewteen anesthetized raster and awake SLS, p = 1E-6 between anesthetized SLS and awake raster, and p = 0.08 between anesthetized SLS and awake SLS.
